# Flash microwave-assisted solvothermal (FMS) synthesis of photoactive anatase sub-microspheres with hierarchical porosity†

**DOI:** 10.1039/d0ra05796g

**Published:** 2020-10-08

**Authors:** M. Davide Cappelluti, Emina Hadzifejzovic, John S. Foord, Duncan H. Gregory

**Affiliations:** School of Chemistry, University of Glasgow University Avenue Glasgow G12 8QQ UK Duncan.Gregory@glasgow.ac.uk; School of Engineering, University of Glasgow Oakfield Avenue Glasgow G12 8LT UK; Chemistry Research Laboratory, University of Oxford Mansfield Road Oxford OX1 3TA UK

## Abstract

The synthesis of nanostructured sub-microspheres of TiO_2_ anatase with hierarchical nano- and mesoporosity was successfully achieved by using an innovative approach that applies the principles of acidic digestion to microwave (MW) solvothermal synthesis. This process, termed flash microwave-assisted solvothermal (FMS) synthesis, facilitates the formation of spherical particles without surfactants or templating agents, exploiting the rapid reaction kinetics engendered by MW heating. Unlike many other MW-assisted solvothermal methods, the application of constant MW power leads to a rapid increase of the autogenous pressure, inducing burst-nucleation of small primary crystallites and subsequent rapid agglomeration into secondary particles, with reaction times reduced to minute-timescales. The use of non-aqueous polar solvents such as ethanol is key to the production of regular spheres with a narrow size distribution, composed of nanocrystallites. Morphology, porosity, specific surface area, phase composition, crystallite size and optical properties of the particles can be controlled *via* a judicious selection of physical and chemical synthesis parameters, especially precursor choice and acid concentration. The complex structure of the particles leads to surface areas of up to *ca.* 500 m^2^ g^−1^ with intergranular mesoporosity. The as-synthesised FMS particles show increased adsorption under dark conditions and selective de-ethylation of rhodamine B under visible light compared to a commercial photocatalyst (Degussa P25). The photodegradation mechanism hinges on the capacity of the spheres to accept electrons from the photoexcited state of molecules at the particle surface, with the large sphere surface area maximising adsorption capacity and improving the efficiency of the photocatalytic processes. The singular characteristics and properties of the particles could pave the way for further applications in water purification and optoelectronic devices.

## Abstract 2

Photoactive nanostructured TiO_2_ sub-microspheres can be fabricated by “microwave flash synthesis” in minute timescales.

## Graphical abstract

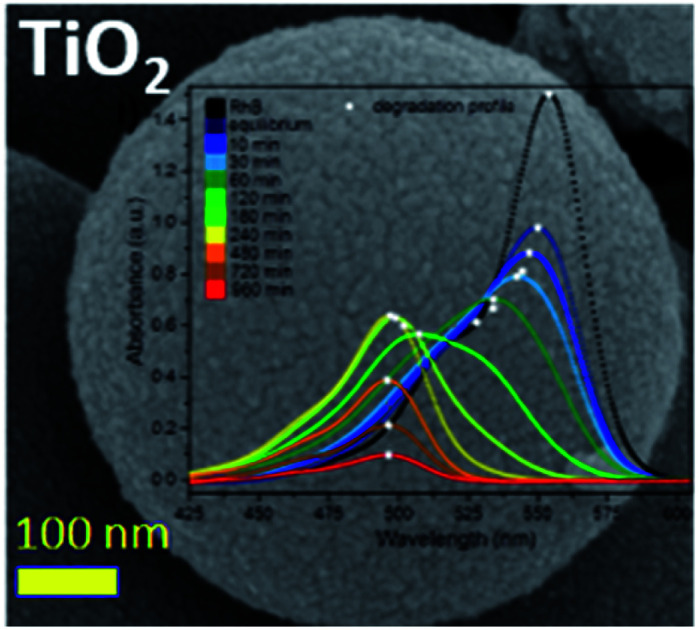

## Introduction

Titanium dioxide (TiO_2_) is one of the most versatile materials available with everyday essential uses (as a pigment/additive in sunscreen, paints and toothpaste)^1–3^ to very specific technological applications in photocatalysis, water splitting and energy conversion devices such as dye-sensitized solar cells (DSSCs).^4^ Due to its low toxicity and high chemical and thermal stability, TiO_2_ has gained favour in environmental applications; notably in gas and water purification.^5^

Microspheres produced by the self-assembly of nanoparticles (NPs) are useful mesoporous materials with hierarchical superstructures.^6^ Their high porosity and tuneable architectures are important for catalysis and sensing applications and increasingly for DSSCs.^7^ Such microspheres are also promising in energy storage and can serve as electrodes for Li ion batteries.^8,9^ Further, hierarchical TiO_2_ microparticles have a future in environmental technologies such as water treatment and purification *via* photo-enhanced mineralisation of organic matter and microorganisms.^10^ Highly photoactive-high surface area mesoporous TiO_2_ microparticles have the potential to replace titania NPs, allaying issues such as the dispersibility and toxicity of semiconductor NPs in the colloidal state.^11–13^ Mesoporous TiO_2_ microparticles can be simply separated from water and the catalyst recovered under gravity, without causing secondary pollution.^13^

Often the main challenge in the use of micron-scale particles is to prevent reduction of surface area and, for example, a corresponding loss of photocatalytic efficiency. One approach is to nanostructure the particles hierarchically, introducing porosity across length scales. Mesopores increase not only the specific surface area but also the density of reactive sites, which are fundamental for photocatalytic applications. Indeed, an improvement of photoactivity can also be achieved in the presence of macropores, which provide a light-transfer path for the distribution of photon energy.^14^

Among methods for the production of TiO_2_ NPs and mesoporous microparticles, solvothermal synthesis yields high purity and crystallinity, both of which are essential in optical and electronic applications. Porosity can be modified in such materials by tempering the reactivity of the Ti(iv) precursor or by employing stabilizing or templating agents such as surfactants and co-polymers.^15,16^ Meanwhile, replacing conventional heat sources in solvothermal reactions with microwaves (MWs) dramatically enhances the reaction kinetics. This can reduce synthesis procedures to one step and can overcome the main limitations of hydrothermal processes by cutting durations from many hours (or days) to minutes.^17^ MW-assisted routes now exist for the synthesis of many inorganic nanomaterials, including TiO_2_ NPs.^18–21^ However, to produce self-assembled, hierarchical structures such as mesoporous microspheres, requires more complicated procedures, typically employing templating agents.^22,23^

In this paper, we propose a “Flash Microwave Solvothermal (FMS)” route to synthesise spherical mesoporous microspheres. The concept of flash chemistry was introduced relatively recently in the wider literature to describe highly controlled reactions performed in minute-, or even second-timescales,^24–26^ whereas the term “flash” was associated in the past with rapid materials syntheses such as MW-assisted or-induced processes.^20,27–29^ The flash process herein, however, concerns acidic digestion under MW irradiation, by analogy to methods used in analytical techniques such as elemental trace analysis *via* ICP.^30^ MW irradiation facilitates the extremely rapid heating of polar solvents in closed systems to temperatures above their boiling points, due to the increase of the autogenous pressure during the heating process.^31^ This can lead to 1000-fold acceleration of reaction rate when using ethanol in a closed MW system, for example.^32^ Acids in solution further contribute to pressure rises in closed systems *via* decomposition processes.^33,34^ Indeed, combining non-aqueous solvents with acidic anions has proved key in the formation of microspheres in such MW syntheses without the need of templating agents.^35–37^

Commercial MW reactors equipped with pressure sensors and capable of controlling the reaction temperature through incident MW power have been successfully employed for MW solvothermal synthesis.^31,37–39^ FMS synthesis differs from this approach, applying constant MW power and exploiting the variation in pressure as the reaction proceeds.^40^

To the best of our knowledge, there are no systematic studies reported for the synthesis of nanostructured sub-micron titania using second-scale MW-assisted solvothermal synthesis, mainly due to the difficulties in controlling the reaction parameters and in observing how these affect the resulting products. Not only were we intrigued to compare the FMS materials to other TiO_2_ in terms of structure and composition, but also we were interested to investigate how certain physical and chemical properties might be influenced by the preparation method and ensuing hierarchical mesoporous structure. Therefore we measured a number of physical properties and focused on photocatalysis as a key chemical property of TiO_2_. The FMS materials were compared both with commercially available TiO_2_ catalysts (Degussa P25) and with lab-scale TiO_2_ microspheres synthesied by established conventional routes.^37,38^ The photodegradation of rhodamine B (RhB) was used as benchmark test for the qualification of the FMS–TiO_2_ microsphere photocatalytic properties, making use of the rich and detailed literature available describing RhB degradation in the presence of TiO_2_ materials.^41,42^

## Experimental methods

### Microwave-assisted flash hydrothermal synthesis

All chemicals were used as received without further purification. Titanium tetraisopropoxide (TTIP, Sigma-Aldrich, >97%) was added dropwise to an acidic solution in an ice-bath to prevent the fast hydrolysis reaction of the alkoxide. The acid, HNO_3_ (Fluka, 70% v/v), was used as a means to control the growth and nucleation process in a series of experiments with a concentration varied from 0.5 to 2 M while varying the concentration of the added alkoxide (20 to 640 mM). Either de-ionised water (Millipore Q-water, 18 MΩ) or absolute ethanol (VWR) were used as solvents. After 30 minutes of continuous stirring, the precursor solution was poured into a 45 mL double-walled autoclave vessel consisting of an inner Teflon container and an outer shell of high strength MW-transparent polymer (MW digestion bomb, Anton-Paar, US). The autoclave was placed into a multimode cavity (MMC) MW reactor (Sharp R272, 800 W). MW irradiation at 800 W, 2.45 GHz was applied for times between 15–180 s. For most synthesis experiments, the irradiation time was set to 60 s.

The resulting cloudy colloidal suspension was centrifuged and washed with de-ionized water and absolute ethanol to separate the precipitate from the acid solution. The precipitate was dried at 60 °C for 2 h to obtain a very fine powder. All samples were sealed and stored in a desiccator to prevent moisture ingress. Selected samples were subsequently calcined at different temperatures (300–600 °C) for 3 h in air for comparison with as-synthesised materials.

### Characterisation techniques

Scanning Electron Microscopy (SEM) was performed with a Philips/FEI XL30 ESEM (20 kV, maximum magnification 20k) equipped with an INCA X-Act detector (Oxford Instruments Analytical, UK) for energy dispersive X-ray spectroscopy (EDS). High-resolution SEM images were collected using a Carl Zeiss Sigma Variable Pressure Analytical SEM (resolution of 2.8 nm/1.5 nm at 1 kV/15 kV) fitted with a Oxford Microanalysis detector. The particle size distribution was estimated by measuring the diameter of hundreds of particles across multiple data sets by using the ImageJ software package (a typical procedure shown in Fig. S1†).^43^ TEM images and SAED analysis were performed by using a FEI Tecnai TF20 microscope (spherical aberration coefficient of 1.2 mm, with 2.4 point resolution and 1.5 line resolution). The instrument was also used to perform high angle annular dark field (HAADF) measurements. X-ray photoelectron spectroscopy (XPS) was performed using a VGX900-Escalab instrument (Kα Al, *E* = 1486.6 eV, operating pressure 1 × 10^−6^ Pa). Depth profile XPS analysis by argon sputtering was performed on selected samples. All XPS spectra were analysed using XPS Casa software. Nitrogen, carbon and hydrogen content was estimated by combustion analysis with a CE440 elemental analyser (Exeter Analytics, UK).

Powder X-ray diffraction (PXD) was performed with a PANalytical X'Pert Pro MPD diffractometer (Cu Kα radiation, *λ* = 1.5406 Å; accelerating voltage and emission current of 40 kV and 40 mA, respectively). PXD patterns were collected at room temperature for 15 ≤ 2*θ* ≤ 85° with a step size of 0.0167° s^−1^. Microstructural characterisation from PXD data was performed by whole powder pattern modelling (WPPM) using PM2K software.^44,45^ IR spectra were collected at room temperature (50 scans/sample, 8 cm^−1^ resolution) using a Shimadzu FTIR 8400S spectrometer with a Pike MIRacle ATR sampling accessory. Raman spectroscopy was performed by using a Horiba-Jobin Ivon LabRAM HR confocal microscope (Horiba Ltd., Kyoto, Japan) system with a variable optical hole aperture (100–300 μm) 600 mm^−1^ grating and a Synapse CCD detector. The excitation source was a Nd:YAG second harmonic laser (Ventus532, Laser Quantum, emission *λ* = 532 nm, output power 50 mW to 1.5 W). The spectra were collected over an effective Raman shift range of 10–1000 cm^−1^. Laser intensity was tuned from 1 to 25% of the maximum power, preventing *in situ* modification (heating) of the samples (*e.g.* phase transformations). Dynamic Light Scattering (DLS) was performed with a Malvern Zetasizer instrument.

The optical band-gaps of samples was determined using diffuse reflectance UV-Vis (DR-UV-Vis) spectroscopy (Shimadzu UV-2700 equipped with an integrating sphere; ISR-2600 Plus, Shimadzu, Japan). BaSO_4_ (Nacalai Tesque, Japan) was used as a reference for both absorbance and reflectance measurements. The spectra were recorded at room temperature in a wavelength range of 190–1300 nm.

Simultaneous thermogravimetric-differential thermal analysis (TG-DTA) was performed with a Netzsch STA 409 PC (Germany) instrument. Mass spectrometry (MS) of the evolved gases during the thermal analysis was performed with a Hiden Analytical HPR 20 instrument, equipped with a quadrupole mass analyser. Nitrogen physisorption experiments for determination of specific surface area and pore analysis were performed using a Quantachrome EVO analyser.

### Photodegradation tests

Rhodamine B (RhB, C_28_H_31_N_2_O_3_Cl, CAS: 81-88-9, MW = 479.02 g mol^−1^, *λ*_max_ = 553 nm, Sigma-Aldrich, ≥ 95%) was used without further purification as a test molecule for photocatalytic measurements. Powdered TiO_2_ samples were mixed with RhB solution (1.5 × 10^−5^ M) to give suspensions of catalyst concentration from 100 mg L^−1^ to 1 g L^−1^, which were sonicated for 10 min to improve the particle dispersion. Each suspension was stirred under dark conditions for at least 30 min to achieve the adsorption–desorption equilibrium of RhB on the particle surfaces. Each suspension was irradiated with different sources of light, under continuous stirring. The temperature was monitored and maintained at 20 °C. The dye concentration was measured *via* UV-Vis absorption spectroscopy (Shimadzu UV 2600 spectrophotometer). The photocatalytic activities of commercial anatase and TiO_2_-based photocatalysts (Aeroxide® P25, Evonik, Germany and KronoClean 7000, Kronos, Germany) were measured following the same procedure for comparison.

## Experimental results

### Structural analysis

**Fig. 1:**
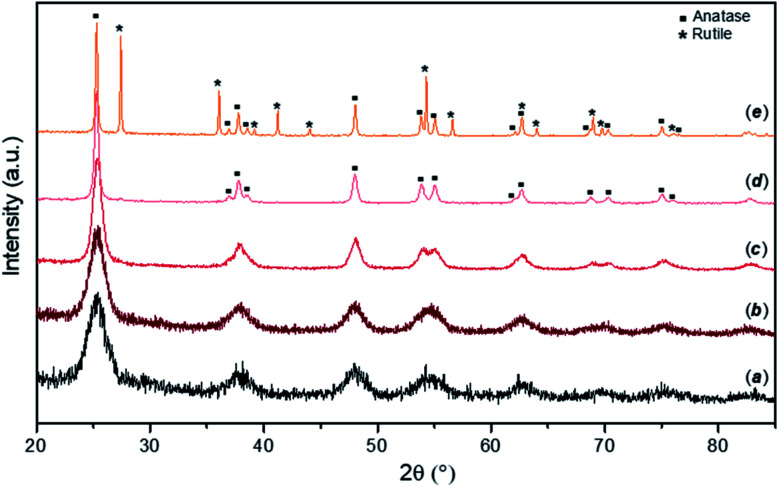
PXD patterns for as-made FMS samples (2 M HNO_3_/160 mM TTIP; 1 min MW treatment) calcined for 3 h in air at different temperatures showing: (a) as-prepared sample; samples after heating to: (b) 300 °C, (c) 400 °C, (d) 500 °C and (e) 600 °C respectively. Anatase (squares) and rutile (asterisks) reflections are indicated on the patterns for samples heated at 500 °C and 600 °C respectively.

Typical PXD patterns of as-synthesised FMS–TiO_2_ samples before and after calcination at different temperatures are presented in Fig. 1. The as-synthesised particles (pattern a) show a low degree of crystallinity, with broad peaks typical for nano-sized crystallites attributable to anatase TiO_2_ (ICDD PDF no. 21-1272). From the appearance of the pattern, amorphous domains cannot be excluded and at least 5% of a sample would be expected to contain crystalline domains for Bragg peaks to be discernible by PXD.^46^ Estimations of the crystallite size of the as-synthesised powder by employing either the Scherrer equation or the WPPM method gave diameters of *ca.* 3–5 nm (results available in Fig. S2, S3 and Table S1, ESI†). These results are comparable with previous data on TiO_2_ prepared by hydrolysis of TiCl_4_ at −20 °C, which gave qualitatively similar PXD patterns from samples that were *ca*. 70–80% amorphous containing NPs 2–4 nm in diameter.^47^ Calcining the FMS–TiO_2_ led to narrower peaks in the PXD patterns and evidently improved the sample crystallinity. The phase transition from anatase to rutile occurred in the range 500–700 °C, with the transition temperature depending on the specific synthesis conditions employed. The estimation of crystallite size at each calcining temperature is reported in Table 1. After calcination at 300 °C, the size of the crystallites remains close to that of the untreated particles (<10 nm). Thereafter, the crystal size increases almost linearly between 400 °C and the phase transition temperature, at which point the anatase grains are progressively converted into rutile, slowing the crystal growth rate (Fig. S4, ESI†).

**Table 1:** Crystallite size (diameter), principal Raman bands and experimental indirect band gaps for selected FMS TiO_2_ sub-microsphere samples (2 M HNO_3_, 160 mM TTIP, 1 min MW treatment) before and after calcination at the indicated temperaures. Bands marked with “*” indicate where Anatase and Rutile Raman peaks overlap

	PXD	Raman					DR-UV-Vis
	Crystallite size^a^/nm	E_g,I_ mode^b^		A_1g_ mode^b^	B_1g_ mode^b^	E_g,III_ mode^b^	Band gap^c^/eV
		Max/cm^−1^	FWHM/cm^−1^	Max/cm^−1^	Max/cm^−1^	Max/cm^−1^	
Untreated	4.3	147.9	22.9	392.7	514.4	640.0	3.38
300 °C	5.1	147.0	19.7	398.0	516.2	641.7	3.32
400 °C	8.8	144.1	16.1	395.9	515.6	638.9	3.28
500 °C	20.0	142.5	13.6	394.7	514.2	637.4	3.22
600 °C	38.4	142.8	10.4	399.0*	516.8*	627.3*	3.03

a Estimated from the Scherrer equation (±0.1 nm).

b ±0.1 cm^−1^.

c ±0.03 eV.

### Morphology, size distribution and internal structure

**Fig. 2:**
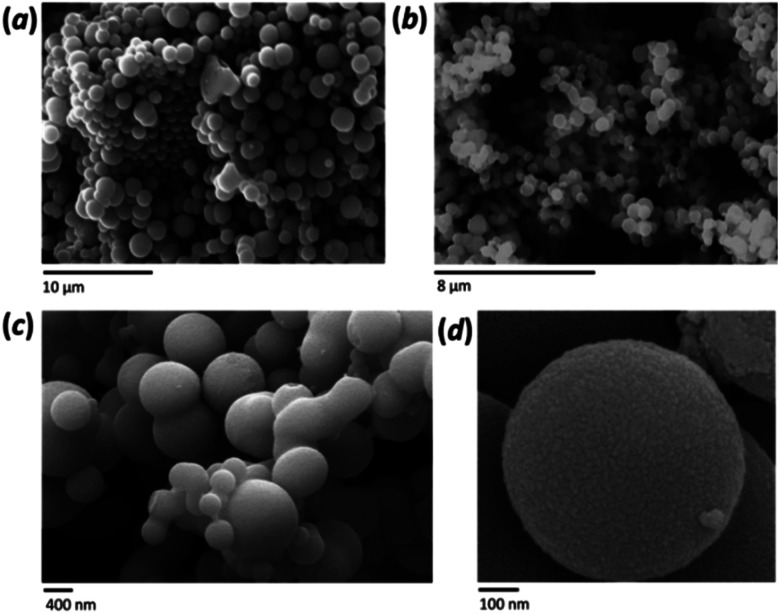
SEM images showing examples of as-made FMS TiO_2_ sub-microspheres using ethanol as a solvent with: (a) 0.5 M HNO_3_/320 mM TTIP; (b) 1 M HNO_3_,/80 mM TTIP. (c and d) HR-SEM images of FMS–TiO_2_ sub-microspheres produced using 1 M HNO_3_,/160 mM TTIP.

A distinctive characteristic of the FMS synthesis is the formation of spherical particles, which become more uniform as they increase in size. The morphology of the sub-microspheres was investigated by SEM analysis (Fig. 2a and b). Both the average diameter and the size distribution of the spheres depends on the synthesis conditions, with the former varying from *ca*. 100 nm to 1–2 μm. Formation of the spherical particles was not observed when an HNO_3_ concentration below 0.5 M was used. Higher resolution imaging confirmed the smoothness of the particle surface (Fig. 2c and d). A size distribution analysis was performed across a tranche of SEM images for each sample and the spheres are relatively monodisperse under most synthesis conditions (with an average relative standard deviation of *ca.* 25%). The mean sphere diameter is, however, strongly influenced by the synthesis conditions (ESI, Fig. S5†) and overall the sphere size increased with the amount of precursor and the decrease of the acid concentration; the latter effect being the more dramatic. Higher acid concentrations also tended to narrow the size distribution, whereas using the minimum concentration necessary to produce spheres generated broad or multimodal distributions (ESI, Fig. S6a†). The influence of the precursor was greater at lower acidity and a clear trend is shown when using 1 M HNO_3_ (ESI, Fig. S6b†). Finally, the morphology of the spheres was not affected by the calcination process (SEM distribution reported in Table S3, ESI;† see also below).

The agglomeration behaviour of the microparticles in water was tested by measuring the hydrodynamic diameter *via* DLS. (ESI Fig. S6†). Multiple DLS measurements were performed on the 2 M HNO_3_/80 mM TTIP and 1 M HNO_3_/160 mM TTIP samples that had been analysed in the solid state using SEM. In both cases the maxima of the DLS size distributions were in good agreement with those obtained from SEM image data. Two clear distinctions could be made when comparing the solution state and solid state size distributions, however: first, DLS did not reproduce the bimodal distribution seen by SEM for the 2 M HNO_3_/80 mM TTIP sample, giving rather a broad monomodal distribution in solution (ESI Fig. S6a†); second although the SEM and DLS distribution profiles were similar for the 1 M HNO_3_/160 mM TTIP sample, the latter yielded a broader distribution with a somewhat higher average particle size in solution (Fig. S6b†). Nevertheless, importantly, the DLS measurements demonstrated that good dispersions of the sub-microspheres in aqueous media could be achieved, with no evidence of aggregation to form larger clusters.

**Fig. 3:**
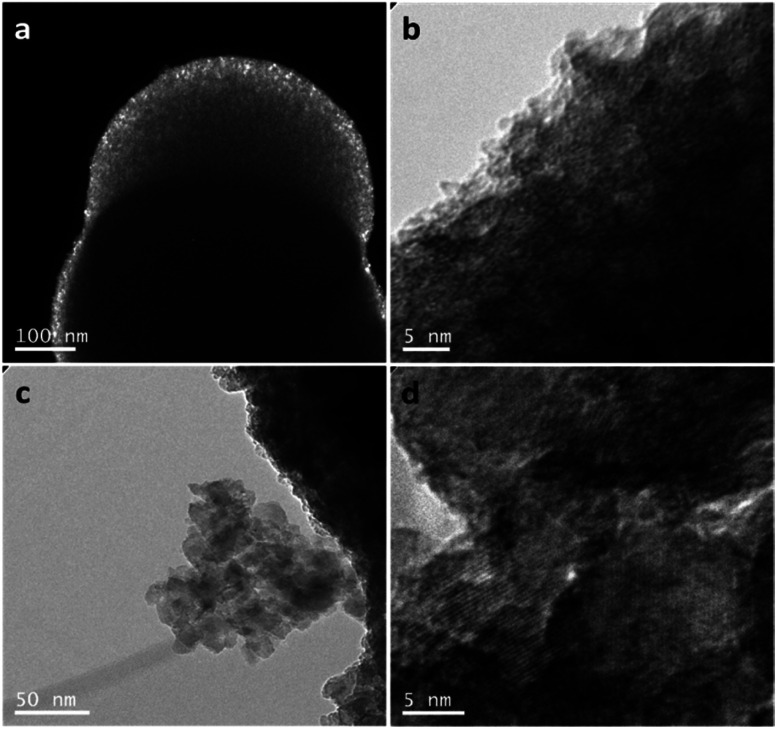
TEM images of as-made FMS–TiO_2_ sub-microspheres synthesised in absolute ethanol with 2 M HNO_3_,/160 mM TTIP: (a) dark field image of the spheres; (b) bright field image showing the detail of the sphere surface at high magnification; (c) cluster of primary NPs dissociated from a sub-microsphere after calcination at 500 °C for 3 h (10 °C min^−1^) and (d) detail of the lattice fringes observable at high magnification after calcination at 500 °C for 3 h (10 °C min^−1^).

Further information about the intricate internal structure of the samples was gleaned from TEM analysis. Entire spheres were too thick for the electron beam to pass through them (Fig. 3a and ESI, Fig. S7a and b†). However, smaller crystallites are observable at the outer surface/edges of the spheres (Fig. 3b). Although the TEM image resolution did not allow a quantitative determination of the size of these crystallites, the component particles are evidently 3–4 orders of magnitude smaller than the spheres themselves and likely within the size regime determined by the analysis of the PXD data. Clear diffraction fringes are not detectable from these very small particles, also supporting observations from our analysis of the PXD patterns. However, HAADF imaging clearly revealed the presence of the small crystallites, which could be identified by the bright contrast spots at different diffraction angles (ESI, Fig. S8†).^48^ The calcination process tends to cause the growth of the component nanocrystallites (Fig. 3c) without altering the spherical shape of the secondary particles (ESI, Fig. S7c and S9†). The crystallites in the calcined samples are apparently randomly oriented and the anatase (101) planes are easily discernible (Fig. 3d). Selected area electron diffraction (SAED) was also performed on a representative TiO_2_ sample (2 M HNO_3_/160 mM TTIP; 1 min MW treatment) before and after calcination at 500 °C. Before calcination, the small primary particles gave patterns containing diffuse powder rings (whereas for a completely amorphous material such discrete rings associated with a family of planes degenerate into a circular halo^49^). The most intense rings could be indexed to the same reflections observed in the PXD patterns. After thermal treatment, the SAED patterns evolved from rings to spots indicating the growth of the particles and increase in crystallinity (ESI, Fig. S10†).

### Raman spectroscopy

**Fig. 4:**
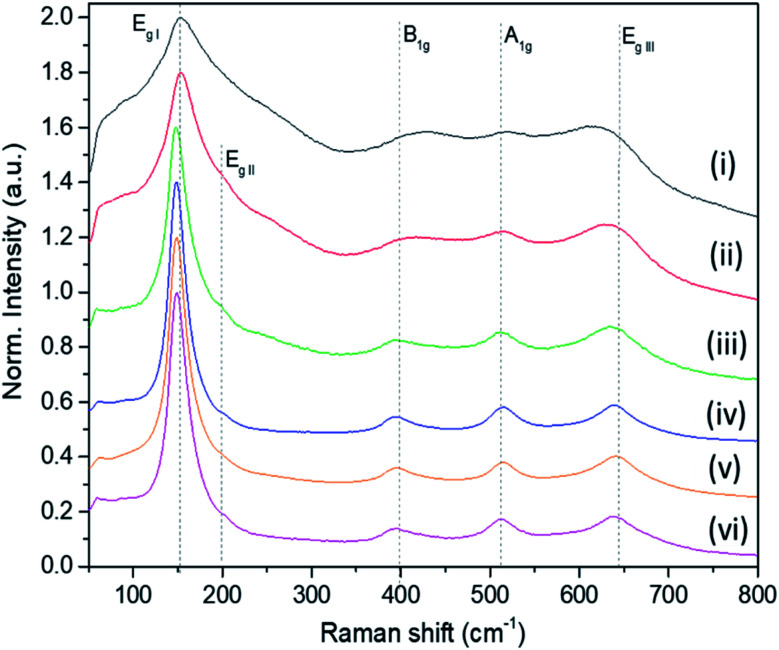
Raman spectra of as-made FMS TiO_2_ sub-microspheres (1 M HNO_3_) as a function of the TTIP concentration, using: (i) 20 mM, (ii) 40 mM, (iii) 80 mM, (vi) 160 mM, (v) 320 mM and (vi) 650 mM TTIP, respectively. The spectra are normalised against the E_g,I_ mode peak intensity.

**Fig. 5:**
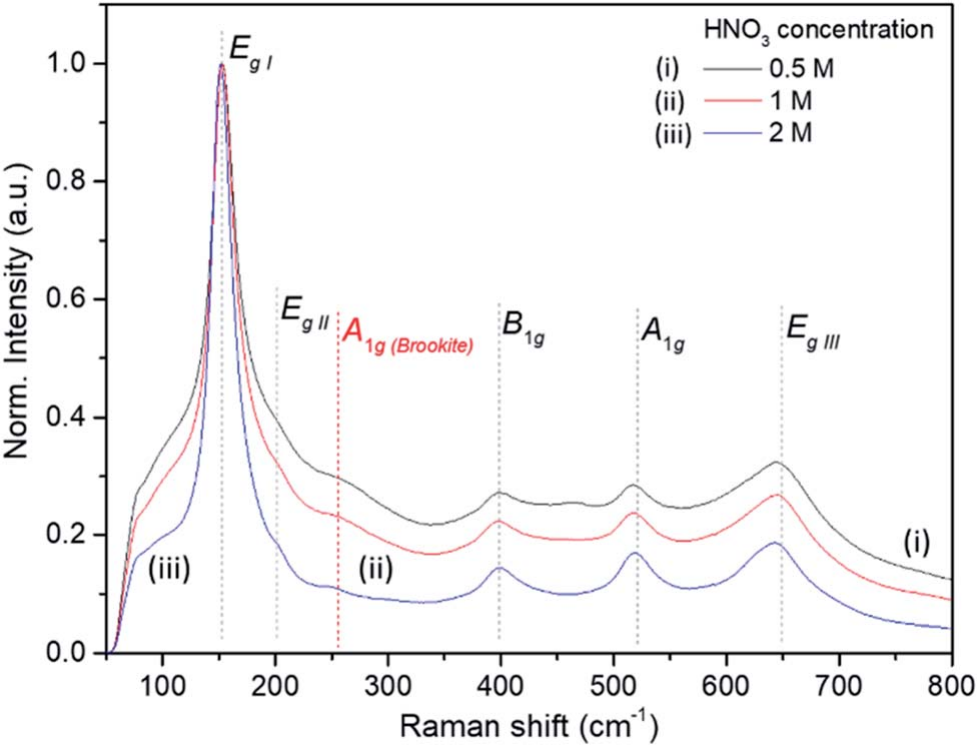
Raman spectra of selected as-made FMS TiO_2_ sub-microspheres (80 mM TTIP) as a function of acid concentration, using: (i) 0.5 M, (ii) 1 M and (iii) 2 M HNO_3_ respectively. The spectra are normalised against the E_g,I_ mode peak intensity.

The Raman bands of the FMS–TiO_2_ particles correspond to those reported for anatase (spectra are shown as a function of acid and precursor concentration in Fig. 4 and 5, respectively). Factor group analysis indicates the existence of 6 Raman active optical modes for the space group (*D*_4h_^19^; *I*4_1_/*amd*) adopted by anatase: E_g,I_ (143 cm^−1^), E_g,II_ (197 cm^−1^), B_1g_ (399 cm^−1^), A_1g_ (513 cm^−1^), B_1g_ (2) (519 cm^−1^, overlapping with A_1g_) and E_g,III_ (639 cm^−.1^).^50^ The definition of the bands is affected by the poor crystallinity of the samples, with absolute intensity comparable to the stray light shoulder (generally occurring before 100 cm^−1^). The E_g,I_ band was shifted to a lower frequency compared to that reported for anatase (Table 1). The full-width at half-maximum (FWHM) of this band is also large, such that it overshadows the E_g,II_ mode. Peak red-shift and broadening can be linked to the presence of nanocrystals, both effects increasing with the decrease of the crystallite size (ESI, Fig. S11†). For very small particles, the quantum size effect leads to the breaking of the rule of phonon momentum conservation for Raman scattering, allowing all phonons present in the Brillouin zone to contribute. The Raman bands are affected by this phonon dispersion, causing broadening and peak shift.^51,52^

Raman spectroscopy further evidences the influence of the synthesis parameters. For example, Fig. 4 shows that increasing the precursor concentration improves the crystallinity of the final particles, with Raman bands demonstrating a reduced shift and less broadening. A similar effect is observed as the acid concentration is increased (Fig. 5; ESI Table S2†). A very small peak at *ca.* 250 cm^−1^ can be observed in less crystalline samples. This peak might be attributed to the A_1g(4)_ mode of brookite, which occurs at 246 cm^−1^ and is one of the few bands that do not overlap with anatase modes (ESI, Fig. S12†).^53^ It is conceivable that a low phase fraction of nanocrystalline brookite could be present in FMS samples given the presence of a very weak and broad signal in the 30 ≤ 2*θ* ≤ 35° region of the PXD patterns, which could correspond to the most intense (121) peak of brookite (2*θ* = 30.83° for Cu Kα radiation).^54^

### Surface area and pore size distribution

**Fig. 6:**
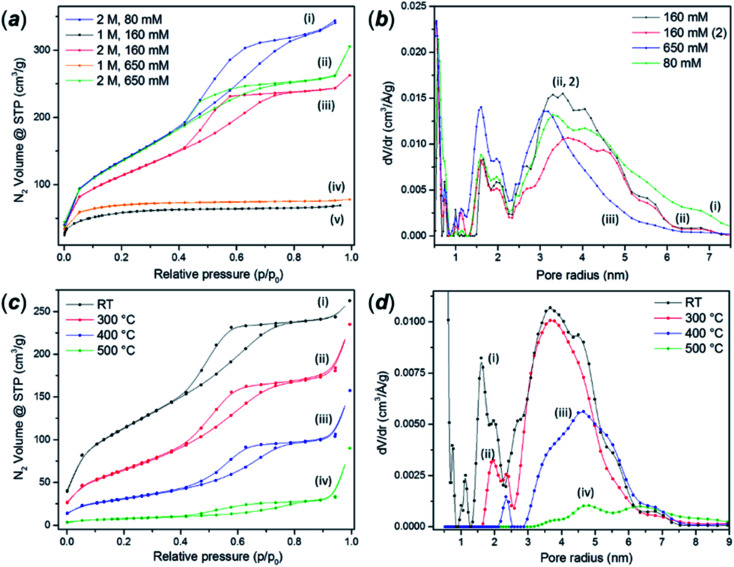
(a) Nitrogen adsorption–desorption isotherms of as-made FMS TiO_2_ sub-microspheres prepared using different concentrations of HNO_3_ and alkoxide precursor respectively: (i) 2 M HNO_3_/80 mM TTIP, (ii) 1 M HNO_3_/160 mM TTIP, (iii) 2 M HNO_3_/160 mM TTIP, (iv) 1 M HNO_3_/650 mM TTIP and (v) 2 M HNO_3_/650 mM TTIP; (b) QS-DFT calculation of the pore size distribution performed on the adsorption branch of selected FMS TIO_2_ sub-microsphere samples showing type IV isotherms, synthesised using 2 M HNO_3_ and: (i) 80 mM TTIP, (ii) 160 mM TTIP and (iii) 650 mM TTIP respectively; (c) nitrogen adsorption–desorption isotherms of mesoporous FMS TiO_2_ sub-microspheres (2 M HNO_3_/160 mM TTIP) after (i) no further treatment or calcination at: (ii) 300 °C, (iii) 400 °C and (iv) 500 °C; (d) QS-DFT calculation of the pore size distribution performed on the adsorption branch of the respective isotherms (i)–(iv) shown in (c).

The as-synthesised FMS TiO_2_ sub-microparticles possess a very high specific surface area. Fig. 6a shows adsorption–desorption isotherms of TiO_2_ particles synthesised using 1 M and 2 M HNO_3_ and different precursor concentrations. BET analysis of the isotherm data of some representative samples gave values from *ca*. 50–500 m^2^ g^−1^ depending on the synthesis conditions (ESI, Table S3†). An increase in acidity leads to a transformation of the isotherms from type II to type IV according to the IUPAC classification. The hysteresis profile of type IV isotherms is characteristic of the presence of mesopores.^55^ The shape of these curves indicates the presence of pore blocking, typical of a mesoporous network with a large size distribution of pore neck width (H2(b) hysteresis loop) and the same hysteresis has been observed in mesoporous ordered silica materials after hydrothermal treatment.^56^ A classical estimation of the pore size distribution for the 2 M HNO_3_ samples using the BJH method gave a pore size distribution centred at a radius of *ca.* 2 nm, the lower edge of the mesopore region in the conventional classification (Fig. 6b) However, DFT analysis revealed a more complex pore network composed of micropores with radii of *ca.* 1 nm, larger cavities with radii between 1.5–2.4 nm and mesopores in the range 2.5–6 nm. The same analyses performed on the microporous samples prepared using 1 M HNO_3_ revealed only the presence of pores between 1–1.8 nm across (ESI, Fig. S13†).

As expected, calcination dramatically reduces the surface area to values comparable to those reported for crystalline bulk anatase. The growth of the crystallites progressively removes the porosity and narrows the hysteresis loops (Fig. 6c). The analysis of the pore size distribution as a function of the calcination temperature (Fig. 6d) confirmed the progressive disappearance of the micropores followed by the mesopores. The micropores likely arise from the voids between crystallites, which diminish as the grains grow, although it is also possible that the smaller cavities could nucleate or combine, shifting the distribution towards larger values before further densification.

### Impurities and surface characterisation

The IR spectra reflect how the surfaces of the as-made FMS sub-microspheres bear some of the functionalities associated with the starting reagents. Analysis of the as-synthesised sub-microspheres (ESI, Fig. S14a†) revealed the presence of hydroxylated surfaces, with broad signals between 3600–3200 cm^−1^ and the band at 1640–1623 cm^−1^ indicating the stretching and bending modes respectively of O–H in water.^57^ Bands at 1222, 1137 and 1048 cm^−1^ could be attributed to *δ*(Ti–OH) vibrations as seen in the presence of strong H-bonding with water molecules such as in TiO_2_ produced by sol–gel methods.^58^ Other bands could be assigned to C=O stretching modes from free carboxylic acid groups (*ca*. 1715 cm^−1^) and Ti–O–C bridging vibrations (1080 cm^−1^) just as have been observed previously in sol–gel-derived TiO_2_ using complexed forms of TTIP as a precursor.^59^ Similarly, the use of HNO_3_ led to further IR modes indicating nitrates (1350 cm^−1^) and nitrites (1260 cm^−1^).^60^ Apart from these decomposition products of HNO_3_, which are generally observed in nitric acid-mediated syntheses,^61^ the presence of adsorbed molecules of the acid itself cannot be excluded, since its strong IR signature at 1711 cm^−1^ in the gas phase is reported to shift to 1677 cm^−1^ when the molecule is adsorbed to a surface.^62^ Not surprisingly, calcination has the effect of removing all the functionalities observed in the as-made materials, with all the respective IR bands absent after treatment at 600 °C (ESI, Fig. S14b†).

XPS analysis provided additional information about the surface composition of the as-synthesised samples (ESI, Fig. S15 and S16†). The characteristic signals of Ti^4+^ species were observed at *ca*. 458 eV (2p_3/2_) and *ca*. 465 eV (2p_1/2_). However, small peaks at lower and higher binding energy (*ca*. 456.9 eV and 460.6 eV) of the main 2p_3/2_ component were also observed in the high resolution Ti 2p spectrum as shown in Fig. S16c.† These have been observed previously and are associated with non-stoichiometric defects, such as Ti^3+^ and Ti^2+^.^63,64^ A broad peak at 529.5 eV is consistent with the O 1s binding energy for O^2−^ in TiO_2_ (528.5–529.7 eV).^65^ The broadening of this peak could be attributed to secondary contributions at 532 eV from adsorbed oxygen on the TiO_2_ surfaces (530.5–533.8 eV).^66^ A quantitative estimation of the C and N-based impurities in the as-synthesised particles was performed by comparing data from combustion microanalysis and depth profile XPS analyses. Combustion microanalyses confirmed the presence of traces of N (*ca.* 1 wt%) and C content ranging from 1.5–3.5 wt% (ESI, Table S4†). XPS depth profile analysis *via* Ar etching of the particle surfaces was performed (Table S5†). After etching to 10 nm, the amount of C is reduced with a concomitant increase in Ti and O. The XPS results are influenced by the contamination of the carbon tape used to prepare the samples. However, the shift of the C 1s peak towards lower binding energies (ESI, Fig. S16a†) and especially the large reduction in the carbon signal during etching shows that samples as synthesised – *i.e.* before etching – contain large amounts of adventitious carbon at the surface. The presence of nitrogen is not observable in the as-synthesised sample because of this carbon contamination, but after etching a small N signal shows the samples are lightly doped (<1%, Table S6†) with N, consistent with the combustion analysis. The high-resolution N 1s signal (ESI, Fig. S16e†) shows a binding energy of *ca*. 400 eV consistent with substitutional nitride species in the oxide lattice.^67^

**Fig. 7:**
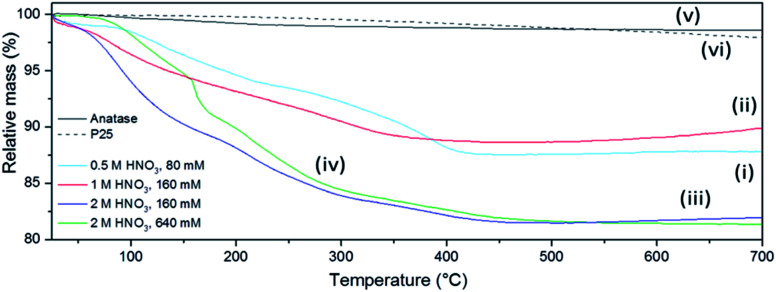
Thermogravimetric (TG) profiles of selected as-prepared FMS–TiO_2_ samples (1 min MW irradiation) synthesised with (i) 0.5 M HNO_3_/80 mM TTIP, (ii) 1 M HNO_3_/160 mM TTIP, (iii) 2 M HNO_3_/160 mM TTIP, (iv) 2 M HNO_3_/640 mM TTIP in comparison to (v) commercial anatase and (vi) Aeroxide® P25 respectively.

The presence of volatile products was evaluated by simultaneous TG-DTA coupled to MS for evolved gas analysis. Fig. 7 shows the mass loss of the sub-microspheres to be 10–20% depending on the synthesis conditions, as compared to negligible mass losses for commercial anatase or P25 under the same conditions. Nearly ¾ of the total mass loss for the sub-microspheres occurred before 300 °C, whereas after 500 °C no further mass loss was detected. This mass loss can be mainly attributed to the release of adsorbed water and unbound acid molecules (up to *ca.* 150 °C) and to structural water (up to *ca.* 250 °C).^68^ The signal of this released water was not incontrovertibly detected by evolved gas MS analysis as it is probably masked by the dominant signal of the carrier gas (Ar). The presence of NO and likely N_2_O could be detected (ESI, Fig. S17†), although the presence of CO_2_ cannot be excluded (given its similar molecular weight to N_2_O). These signals are therefore consistent with IR data and HNO_3_ decomposition products, which have been previously reported to desorb within the observed temperature range.^68^

### Optical properties

**Fig. 8:**
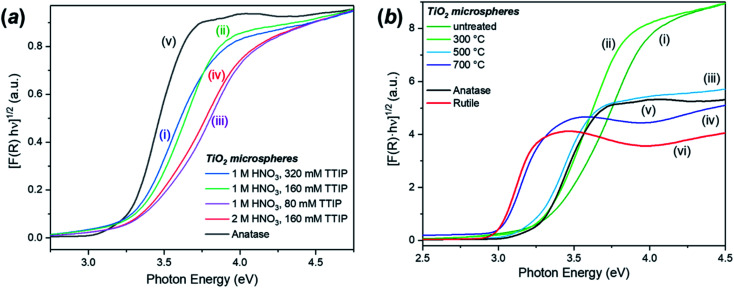
Kubelka–Munk plots for the calculation of the indirect band gap for: (a) selected samples of as-made FMS TiO_2_ sub-microspheres prepared using: (i) 1 M HNO_3_/320 mM TTIP, (ii) 1 M HNO_3_/160 mM TTIP, (iii) 1 M HNO_3_/80 mM TTIP and (iv) 2 M HNO_3_/160 mM TTIP respectively as compared to (v) commercial TiO_2_ anatase and (b) a representative sample of FMS TiO_2_ sub-microspheres (2 M HNO_3_/160 mM TTIP): (i) as-made and calcined at (ii) 300 °C, (iii) 500 °C, (iv) 700 °C, respectively. The plots are compared to those for commercial samples of (v) anatase and (vi) rutile TiO_2_.

**Fig. 9:**
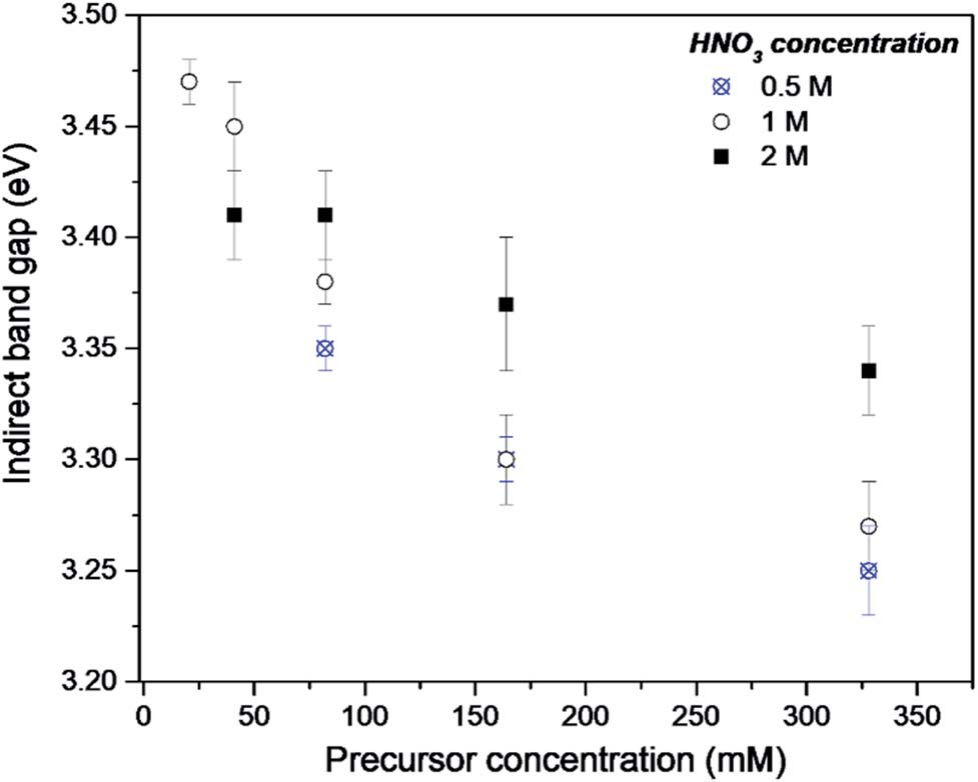
Indirect band gap values for as-made FMS–TIO_2_ sub-microspheres as a function of TTIP concentration using 0.5 M (crosses/circles), 1 M (open circles) or 2 M (filled squares) HNO_3_ respectively.

The DR-UV-Vis analysis of the as-synthesised FMS–TiO_2_ showed a variation in band gap compared to bulk crystalline anatase and the shape of the absorbance spectrum (and the associated Kubelka–Munk functions) was notably different when compared to that of the commercial anatase reference sample (Fig. 8). Calculation of indirect and direct band gaps across FMS samples consistently gave values of *ca*. 3.3–3.4 eV and 3.6–3.8 eV respectively (ESI, Table S6†), which are significantly larger than the values obtained for anatase (indirect band gap of 3.23 eV, direct band gap of 3.47 eV). A closer study of the correlation between band gap and synthesis conditions indicated that increasing the precursor concentration tends to decrease the band gap systematically. However, a correlation of band gap with acidity is not so clear, although there is a general trend of increasing band gap with increasing acidity (Fig. 9 and ESI, Fig. S18†). One possible rationalisation relates to correlations of band gap with crystallite size. An increase in precursor concentration leads to a decrease in crystallite size (as is apparent from analysis of the E_g,I_ mode in Raman spectra, for example). An opening of the band gap is expected from quantum confinement effects for particles < 5 nm in diameter.^69,70^ A higher concentration of precursor molecules might be expected to generate a large number of nuclei, leading to smaller crystallites and particles.^71,72^ However, in the acidic reaction environment under MW heating, such competition can be suppressed and agglomeration of the seed crystallites to minimise the energy of the system, would occur.^73^ The band gap would consequently decrease with precursor concentration under such conditions. The trends above are consistent with our observations from PXD, Raman spectroscopy and SEM/TEM where the primary and secondary structures of the FMS–TiO_2_ materials (*i.e.* both the sub-microspheres and their component nanocrystallites) increase in size with increasing precursor concentration, while overall they tend to decrease in size with increasing acid concentration (ESI, Fig. S5†).

The increase in band gap for the FMS–TiO_2_ materials when compared to commercial anatase is a further indicator of the presence of nanocrystallites in the spheres. Fig. 8b (with data also reported in Table 1) shows how calcination at increasing temperatures (leading to increasing crystallite size) reduces the band gap. The DR-UV-Vis spectra become increasingly similar to that of bulk anatase with higher calcination temperature until ultimately at 700 °C the spectrum resembles that of bulk rutile, with a comparable (reduced) band gap.

### Photocatalytic tests

The photocatalytic behaviour of the FMS materials was evaluated in the aqueous phase by considering the controlled degradation of rhodamine B (RhB). The dye is very stable in aqueous solution without the presence of a photocatalyst, when irradiated with either UVA or visible light. A universal characteristic of the photodegradation experiments was the immediate decrease in the RhB concentration prior to irradiation, during the induction period of the experiment (sonication/stirring in the absence of light to improve the dispersion of the catalyst in the suspension). The dramatic *ca*. 80% drop in dye concentration indicated adsorption of the dye molecules on the surface of the spheres. By comparison, P25 gave only a *ca*. 2–3% decrease in dye concentration during the induction period (ESI, Fig. S19†). The colouration of the FMS TiO_2_ samples clearly indicated a strong adsorption of the dye by the particles. The enhanced proclivity for adsorption is readily rationalised by the remarkable surface area (200–500 m^2^ g^−1^) of the sub-microspheres as compared with values typically an order of magnitude lower for P25 (*ca*. 50 m^2^ g^−1^).^74^

**Fig. 10:**
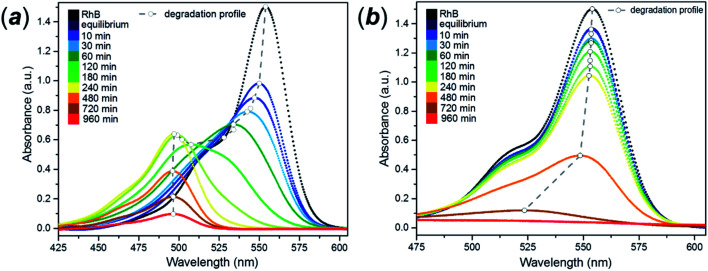
Rhodamine UV-Vis absorbance spectra using: (a) as-made FMS TiO_2_ sub-microspheres (2 M HNO_3_ conc. and 320 mM precursor conc.) and (b) P25 as a catalyst for its degradation under visible light. The dashed trend line indicates the shift in the maximum of the principal peak in each spectrum.

The as-synthesised spheres showed no evidence of photodegradation under UVA light (ESI, Fig. S20†), whereas under visible light a continuous change in the colour of the solution occurred from purple to yellow (ESI, Fig. S21†) Generally, dye degradation using TiO_2_-based catalysts (*e.g.* Aeroxide® P25) is manifested by a decolouration of the original solution, with a fade in colour with exposure time. UV-Vis absorption spectra taken for samples of RhB/FMS–TiO_2_*vs.* time during the experiment showed an hypsochromic shift of the maximum absorbance peak to lower wavelength (from *λ* = 554 nm to 498 nm; Fig. 10a) simultaneous with a reduction in peak intensity. Consistent with visual observations of the corresponding experiment, no such hypsochromic shift occurs for dye degradation using P25 until just before the end point, when a small shift to 525 nm is observed (at which point the concentration of rhodamine is barely detectable and the solution is transparent; Fig. 10b). After 3 h calcination in air at temperatures of 400–600 °C, the TiO_2_ sub-microspheres showed improved photocatalytic activity under UVA light but lost all trace of photoactivity under visible light (ESI, Fig. S22†).

## Photodegradation mechanism

### Selective *N*-deethylation of rhodamine

**Fig. 11:**
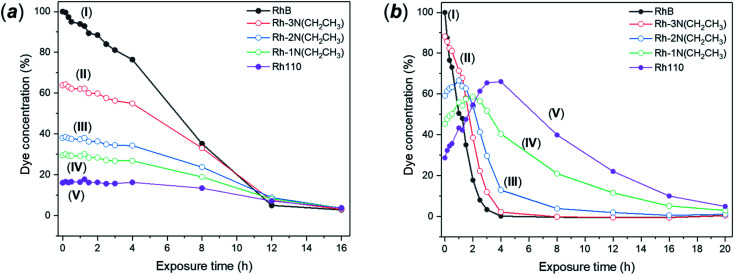
(a) Degradation curves for the 5 degrees of *N*-ethylation of the Rhodamine molecule (where (I) RhB contains 4 *N*-ethyl groups and (V) Rh110 contains none) for: (a) P25 and (b) as-made FMS TiO_2_ sub-microspheres under visible light irradiation; (I) RhB, (II) Rh–3N–Et, (III) Rh–2N–Et, (IV) Rh–1N–Et, (V) Rh110.

Photodegradation of RhB can be proceed *via* two different and independent mechanisms; the direct degradation of the chromophoric conjugated system or the de-ethylation of the four ethyl groups. Direct degradation typically occurs in conventional photocatalytic processes. De-ethylation has been described as an indirect photocatalytic process, as it can occur side by side with the chromophoric cleavage, but it requires a direct chemical contact between the catalyst surface and the dye molecule, allowing the transfer of an electron to the conduction band of the photocatalyst. According to the proposed mechanism, the *N*-ethyl group of the dye provides a free electron pair necessary for the chemisorption.^75^ While in many catalytic degradation experiments the two processes occur simultaneously, the visible light degradation of RhB in the presence of FMS–TiO_2_ appears to follow a selective de-ethylation mechanism until the formation of a product similar to 3,6-diamino-9-(2-carboxyphenyl) chloride, known also as rhodamine 110,^76^ occurs with an absorbance peak maximum at 498 nm. The intermediate de-ethylated fragments present absorbance peaks at 539 nm, 522 nm and 510 nm.^77^ The sequential *N*-de-ethylation of the original molecule causes colour changes until the end of the process when the fully de-ethylated form of rhodamine (Rh110) undergoes the cleavage of the conjugated xanthene unit, ultimately leading to the fading of the solution.^78^Fig. 11 presents the P25 and FMS–TiO_2_ degradation experiments from the perspective of the different degrees of *N*-ethylation of RhB, by considering the intensity of peaks centred at the absorption wavelengths of each of the reported “intermediate” de-ethylated rhodamine molecules (*i.e.* in which 1, 2, 3 or 4 ethyl groups are removed). When using P25 (Fig. 11a), the degradation behaviour for each of the de-ethylated decomposition products is rather similar, with the concentrations decreasing uniformly with time. Conversely, for FMS–TiO_2_ (Fig. 11 b) the tri- and di-ethylated molecules are formed and removed extremely rapidly, followed by the relatively sharp degradation of the mono N-ethylated intermediate and the de-ethylated molecule (Rh 110).

Given that no meaningful hypsochromic shift was observed in the degradation experiment performed in the presence of P25, observation of the absorption spectra (and solution colour) leads one to speculate about the predominance of the cleavage mechanism over de-ethylation. In the presence of FMS–TiO_2_, the initial de-ethylation and the cleavage proceeded at different rates, with the latter mechanism prevailing only on the completion of the former. A similar degradation pathway has been reported for nanocrystalline anatase TiO_2_ by Fan *et al.*^79^ The authors reported how a low degree of crystallinity, high surface area (over 200 m^2^ g^−1^) and crystallite diameters < 10 nm led to predominance of *N*-de-ethylation over chromophoric cleavage, but no further explanation of the phenomenon could be offered at the time. The above behaviour coupled with the lack of FMS–TiO_2_ UVA photoactivity suggests that the dye degradation mechanism bears analogies to that which occurs in the presence of vanadyl ions. According to this mechanism, the FMS–TiO_2_ particles would act as electron acceptors, with photodegradation induced by the excitation of the dye in solution rather than by photogeneration of electrons and holes in the particles. In this situation, the catalyst acts as an electron-transfer mediator, with electrons in the conduction band reacting with oxygen adsorbed on the catalyst surface forming radical species, such as O^.^, OOH^.^ and HO^.^, where the last of these is responsible for advancing the degradation, particularly *via* chromophore cleavage.^80^

Hypsochromic shifts have indeed been observed in single-electron transfer process such as the reduction of VO_2_^+^ under visible light irradiation. The prevalence of *N*-deethylation over chromophore cleavage can be understood in terms of the passage of an electron from the excited state of RhB to VO_2_^+^ (*i.e.* containing V in the +5 oxidation state; pervanadyl). The reduced vanadium species (VO^2+^, vanadyl) are incapable of further electron trapping, hence showing no activity. The same study reported an absence of de-ethylation under UV light when a carboxyl moiety is present on the aromatic molecule.^81^ In this case, the electron transfer would occur preferentially with species able to coordinate with the carboxyl, such as Pt(iv).^82^

### Effect of the structure

Brookite can be also involved in dye photodegradation under visible light and the presence of brookite nanocrystals in FMS–TiO_2_ may be significant in the degradation mechanism. Although the photocatalytic behaviour of brookite as compared to anatase is still under debate,^83^ junctions between the two TiO_2_ polymorphs have been identified as enhancing the photoactivity when compared to the single phases.^83,84^ Brookite has a slightly larger band gap than anatase and the band energy matching of the two polymorphs facilitates the interfacial migration of photoinduced electrons from brookite to anatase.^85^ The junctions act as one-way valves for the charge carriers, to some extent inhibiting their recombination.^82^ However, the behaviour of such brookite–anatase junctions can apparently be very interface-dependent and the possibility of electron–hole pair recombination is often relatively high in a random phase mixture, for example, which leads in turn to inefficient charge separation and a consequent deterioration of photocatalytic and photoelectrochemical properties.^86,87^

Considering the lesser photocatalytic performance of the as-synthesised FMS–TiO_2_ samples under UVA light, it is possible that the presence of amorphous domains in the particle structure is responsible for this behaviour. The reduced photocatalytic performance of amorphous *vs.* crystalline TiO_2_ is generally attributed to a high concentration of defects, which enhance the electron–hole recombination rate.^88^ Higher crystallinity and a higher concentration of larger anatase crystallites, such as is achieved by the calcination of the FMS–TiO_2_ samples, should therefore enhance the photoactivity under UVA light. Conversely, the visible light photoactivity of the FMS samples would be expected to decrease on sintering, not least since an increase in crystallite size leads to the bulk recombination of charge carriers as the diffusion path length for the charges to migrate to the surface increases.^89^ The loss of photoactivity could be also related to the reduction in surface area and the absence of available sites for electron transfer. Although the surface area of some calcined samples remains higher than P25, other factors may affect the degradation mechanism. For instance, the stability of the dispersions of FMS samples in aqueous solution decreased with increasing calcination temperature, with a zeta potential shifting towards progressively less-negative values (ESI; Table S7†). The poorer dispersion stability and lower adsorption capacity of cationic organic pollutants close to neutral pH are detrimental for the photocatalytic performance of the particles.^90^

### Adsorption and sensitisation mechanism

The observation of the purple coloration of the FMS–TIO_2_ at the end of the degradation experiment reinforces the importance of the surface adsorption in the degradation mechanism. The high specific surface area of the spheres leads to higher initial adsorption (*ca.* 20%) during the equilibration phase compared with P25. Similar values of initial adsorption have been reported previously for TiO_2_ particles with rather similar physicochemical properties.^79^ The enhanced initial adsorption of the spheres presents further evidence for the nature of the degradation mechanism. Assuming the spheres possess a slightly negative surface charge in water at neutral pH (the p*K*_a_ of TiO_2_ is estimated to be *ca*. 5.5–6.5),^91^ then the carboxyl moieties of RhB molecules, with a p*K*_a_ of 3.22,^92^ will be deprotonated, hence repulsion between the spheres and the Rhodamine occurs.^93^ The electrostatic repulsion is mitigated by the high surface area of the FMS–TiO_2_, whereas the surface of P25 is not able to compensate the effect. The phenomenon could also explain the charge transfer process involved in the de-ethylation, since the molecule could adhere to the TiO_2_ surface preferentially through the positively charged nitrogen atoms of the *N*-ethyl moieties. The selectivity of degradation *via* de-ethylation *vs.* chromophoric cleavage has been reported to depend on the pH of the RhB solution when using either Pb_3_Nb_4_O_13_ pyrochlore supported on fumed silica^94^ or a TiO_2_/SiO_2_ composite,^95^ with electron transfer facilitated by the molecule orientation.

The process of electron injection into the semiconductor conduction band is well-known for photoexcited dyes and is an underpinning principle in the design of dye-sensitized solar cells (DSSCs) in which a semiconducting oxide (commonly TiO_2_) is sensitised by dye adsorption.^96^ From the evident colour of the spheres at the end of RhB degradation, the RhB molecules adsorbed to the TiO_2_ particle surface remain undegraded. The adsorption phenomenon could thus be understood in terms of a dye-sensitisation, in which the presence of the dye enhances the electron transfer and the degradation process of other molecules in solution.

## Conclusions

Flash microwave-assisted solvothermal (FMS) synthesis is an efficient new method for the ultra-fast preparation of nanostructured, phase-pure mesoporous TiO_2_ spherical sub-microparticles. The spherical particle diameter can be tuned from 2 μm to as small as 200 nm by manipulation of the synthesis parameters (*e.g.* acid and precursor concentration). The particles possess a complex hierarchical structure of nanocrystallites (of the order of 10 nm or less as indicated by multiple characterisation techniques and property measurements) with an exceptionally high surface area (200–500 m^2^ g^−1^) and predominant mesoporosity. The particles form within a narrow size distribution even after only 1 minute of MW treatment and prove to have high dispersibility in aqueous media. This attractive combination of features enhances RhB adsorption and has profound effects on the subsequent photodegradation process. Unlike P25, which by contrast preferentially degrades RhB by chromophoric cleavage, the as-synthesised sub-microspheres exhibit rapid and selective degradation *via N*-de-ethylation. Calcination destroys the visible light activity of the spheres as the porosity and surface area are lost. However, one cannot completely rule out the significance of the surface “impurity” species present in the as-made FMS–TiO_2_ prior to calcination. Further experiments which can decouple the potential surface functionalisation chemistry from the structural characteristics of the TiO_2_ spheres themselves should shed further light on the mode by which the degradation reactions proceed and how one might improve the FMS–TiO_2_ catalytic properties further. Moreover, the structural attributes of the FMS–TiO_2_ spheres and the ease with which they can be made, paves the way for further advances in a myriad of titania-related applications. In fact, the scope could be immense if the FMS technique can be applied more widely in the synthesis of mesoporous inorganic oxides.

## Conflicts of interest

There are no conflicts to declare.

## Supplementary Material

RA-010-D0RA05796G-s001
