# Supplementary material for: Flash microwave-assisted solvothermal (FMS) synthesis of photoactive anatase sub-microspheres with hierarchical porosity
Source: RSC Adv. 2020 Oct 8;10(61):37233–45. doi: 10.1039/d0ra05796g (PMC9057126; doi:10.1039/d0ra05796g)
Supplement: RA-010-D0RA05796G-s001 [file RA-010-D0RA05796G-s001.pdf]

Electronic Supplementary Material (ESI) for RSC Advances.
This journal is © The Royal Society of Chemistry 2020

Supporting Information

Flash microwave-assisted solvothermal (FMS) synthesis of photoactive anatase sub-microspheres with hierarchical porosity.

M. Davide Cappelluti,[a,b] Emina Hadzifejzovic,[c] John Foord [c] and Duncan H. Gregory [a] *

a. *WestCHEM, School of Chemistry, University of Glasgow, University Avenue, Glasgow G12 8QQ, UK*
b. *School of Engineering, University of Glasgow, Oakfield Avenue, Glasgow G12 8LT*
c. *Chemistry Research Laboratory, University of Oxford, Mansfield Road Oxford OX1 3TA, UK*

Further details concerning the experimental methods

The size distribution of particle was calculated by measuring the particle diameters from different SEM images at different magnification. As a standard method, at least 100 particles from 5 different image frames were measured for each single sample. The calculation was performed with the software ImageJ (National Institutes of Health, US).

Raman spectroscopy was performed by using laser intensities from 1 - 25 % of the maximum power in order to avoid *in situ* modification (e.g. heating) of the samples (e.g. phase transformations). Raman data were analysed excluding the effect of the Rayleigh scattering and stray light, cutting the spectral region below 75 cm^{-1}. Baseline subtraction was then performed by using asymmetric least squares smoothing (with an asymmetric factor of 0.001, a threshold of 0.001 and a smoothing factor variable between 3 and 5 depending on the peak resolution). Baseline subtraction was performed using Origin Pro 2016. Raman peak analysis was performed by using the Fytik software (vs 0.98). Taking the asymmetry between the two sides of the peaks into consideration, the $E_{g\,I}$ peak was fitted with a split-pseudo-Voigt function. The function provided a better fit compared to a conventional pseudo-Voigt alternative or to a combination of multiple Lorentzian-Gaussian functions. The other modes were identified through analysis of the first derivative of the spectra.

Optical band gap calculations were performed using the Kubelka-Munk function F(R):

$$[F(R)\cdot hv]^n = \left[\frac{(1-R)^2}{2R}\cdot hv\right]^n$$

where *R* is the absolute reflectance and *hv* the photon energy. The intersection with the *x*-axis of the function plotted against the photon energy gives the value of the optical band gap. The exponent n depends on the nature of the optical transition, adopting n = ½ for indirect and n = 2 for direct transitions respectively.

The specific surface area (SSA) was calculated using the BET equation in the interval $0.05 \le (p/p0) \le 0.33$. The pore size distribution for mesoporous samples was evaluated by implementation of the BJH equation and DFT calculations, assuming a non-linear approach on the equilibrium isotherms and assuming mixed shape for the pores on a carbonaceous matrix.

The dye concentration before and during the degradation experiment was calculated from the absorption intensity by using a calibration curve based on the linear correlation between concentration and absorption intensity described by the Beer-Lambert law.

Figures and tables

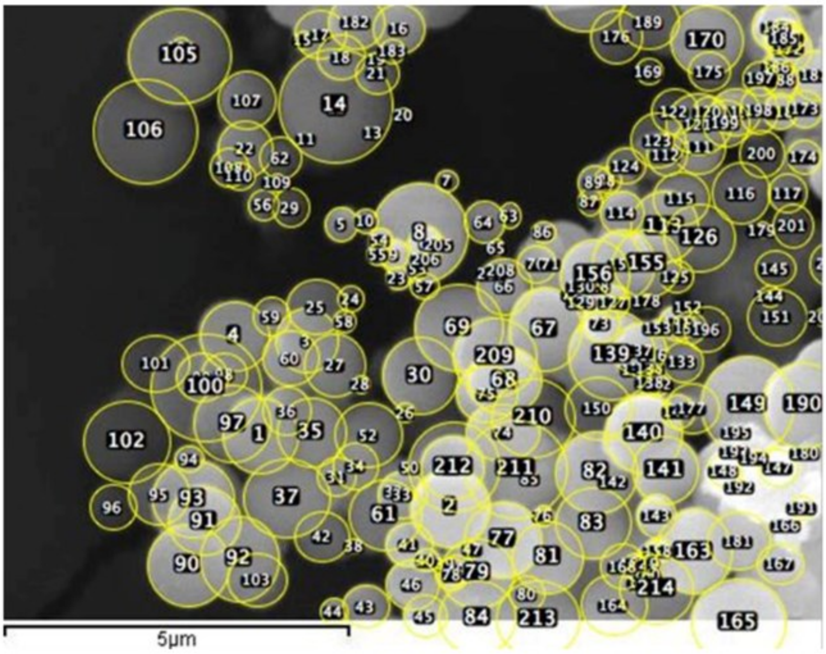

Figure S1: Example of SEM image processing for the identification of the particle size distribution by using the ImageJ software. The diameter of all circled particles is calculated from the area, assuming perfect spherical shape for each encircled particle.

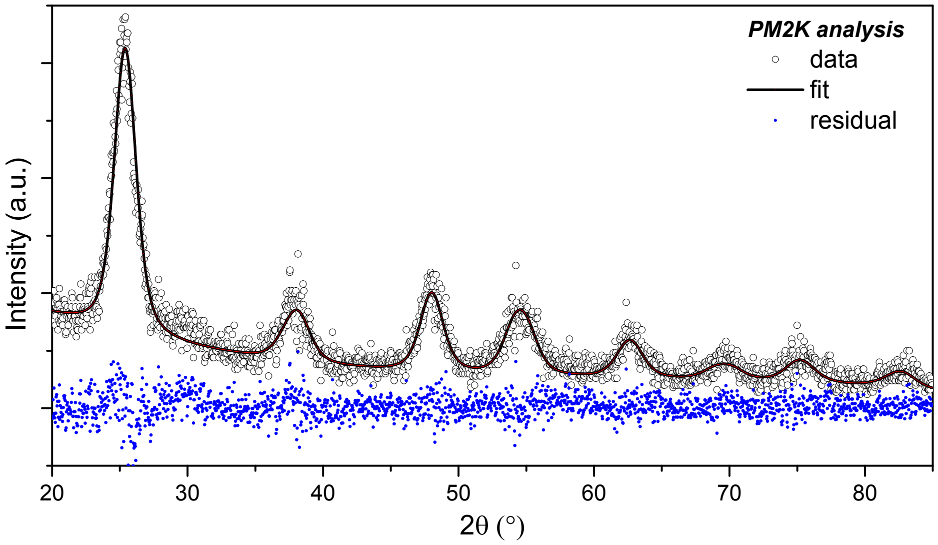

Figure S2: Profile fitting of a sample of FMS-TiO_2 particles (2M HNO_3/160 mM TTIP) using the PM2K software package.[1]

[1] M. Leoni, T. Confente, P. Scardi, Zeitschrift für Kristallographie Supplements, 2006, **23**, 249.

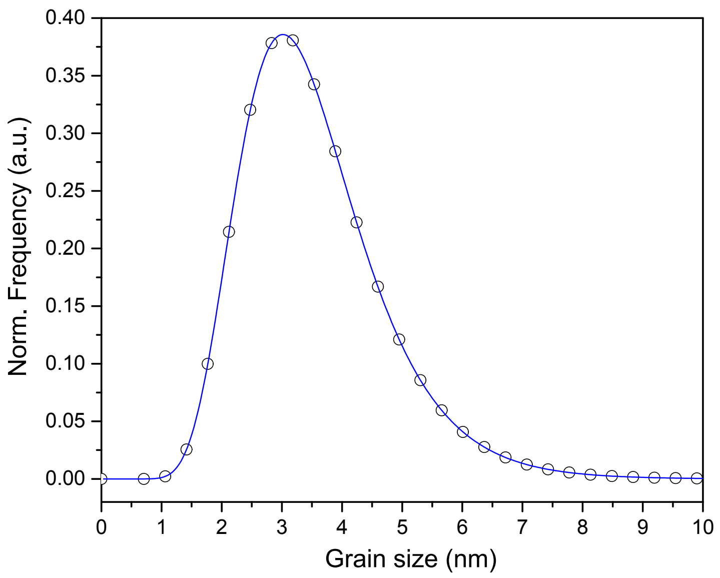

Figure S3: Example of the log-normal distribution of the grain size (diameter) estimated using the WPPM method for FMS-TiO_2 (2 M HNO_3/160 mM precursor concentration; 1 min MW treatment).

Table S1: Results of the WPPM refinement and size analysis compared to results obtained from applying the Scherrer equation.

**FMS-TiO_2,** NE series (2 M HNO3, 160 mM TTIP, 1 min MW treatment)	
2θ (101) (°)	25.292 ± 0.017
FWHM (°)	1.876 ± 0.017
Scherrer's average grain size (nm)	4.34 ± 0.04
**WPPM calculation**	
WPPM distribution peak (nm)	3.35 (3)
WPPM distribution width (nm)	0.33 (4)
**Lattice parameters**	
a (Å)	3.7974(1)
c (Å)	9.4935(94)
**Fitting parameters**	
μ	1.21(8)
σ	0.32(4)
No. of variables	46
No. of observation	1969
R_{wp} (%)	16.7
R_p (%)	18.6
X^2	0.81
GOF	0.905

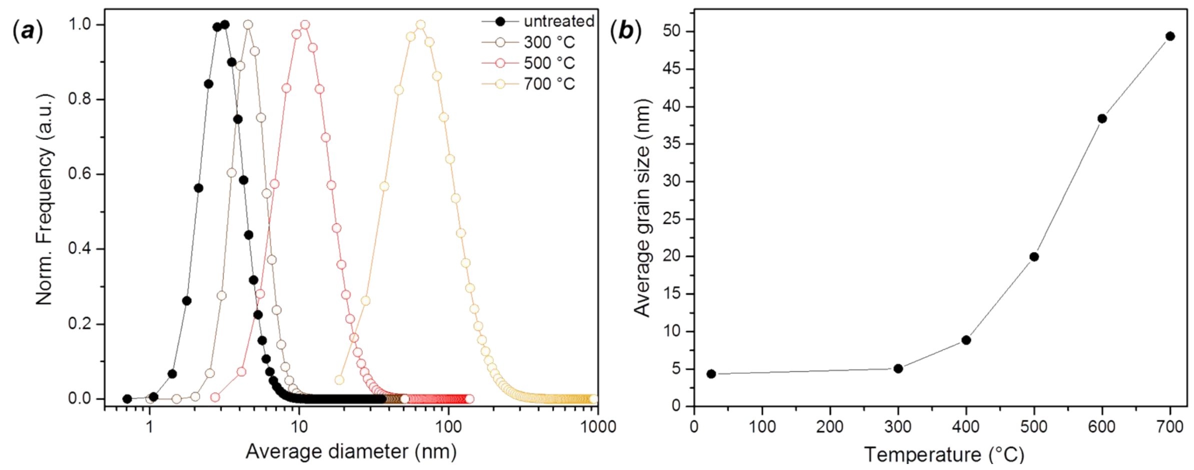

Figure S4: Representations of: (a) the lognormal distribution of the crystallite diameter and (b) the average grain size obtained by the WPPM method for a selected sample of FMS TiO_2 sub-microspheres (2 M HNO_3 concentration/160 mM precursor concentration) as a function of calcination temperature.

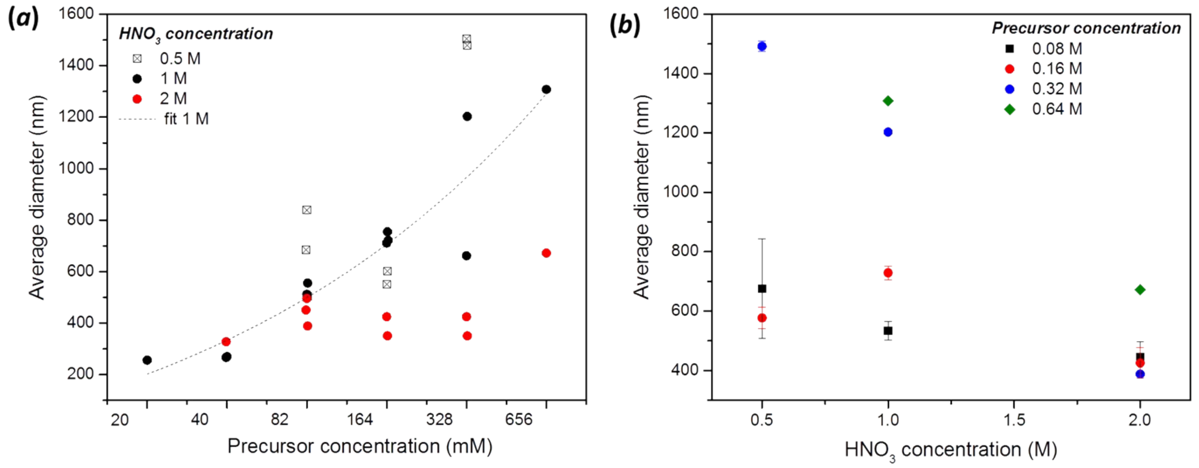

Figure S5: Average diameter of selected FMS TiO_2 sub-microspheres (from SEM data) as a function of: (a) the precursor concentration and (b) the acid concentration.

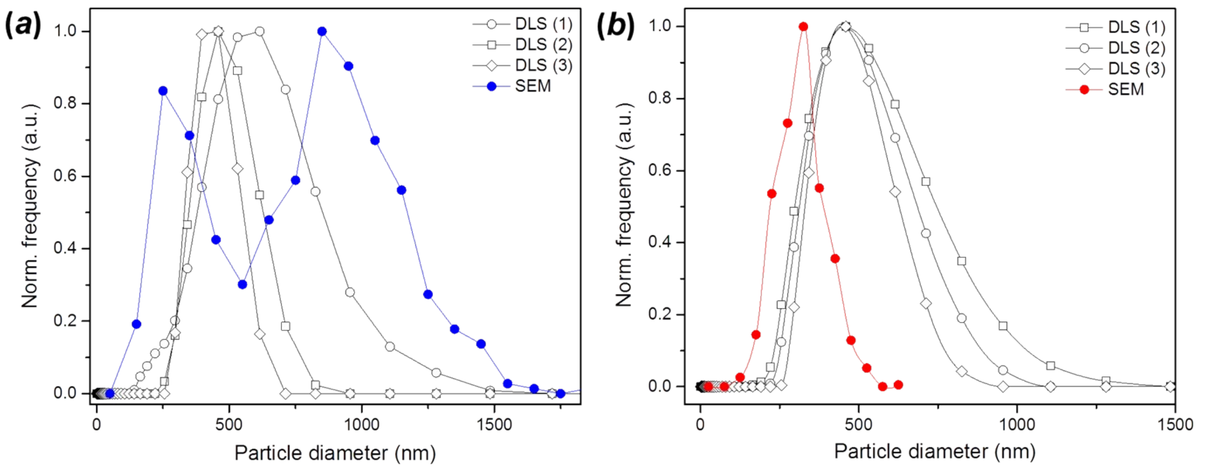

Figure S6: Comparison of particle size distributions obtained from SEM particle counting and DLS measurements for different FMS TiO_2 sub-microsphere samples; (a) 2 M HNO_3/80 mM TTIP concentration samples giving variations in distributions by DLS and a bimodal distribution by SEM; (b) 1 M HNO_3/160 mM TTIP concentration samples giving consistent distributions by DLS and a monomodal particle diameter distribution by SEM measurements.

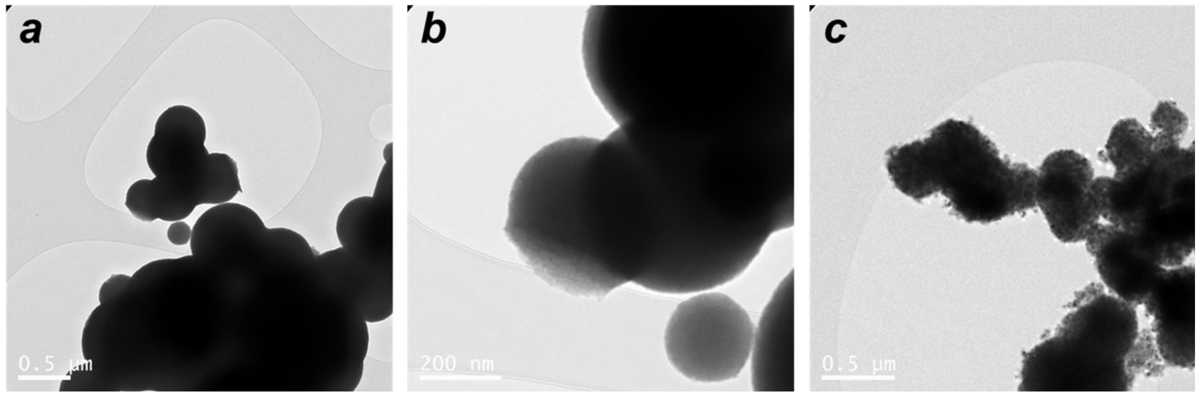

Figure S7: TEM images of FMS-TiO_2 spheres (2 M HNO_3 solution in ethanol/162 mM of TTIP as precursor; 1 min MW treatment): (a),(b) as-synthesised and (c) after calcination treatment (500 °C , 3 h treatment, 5 °C min^{-1} heating rate).

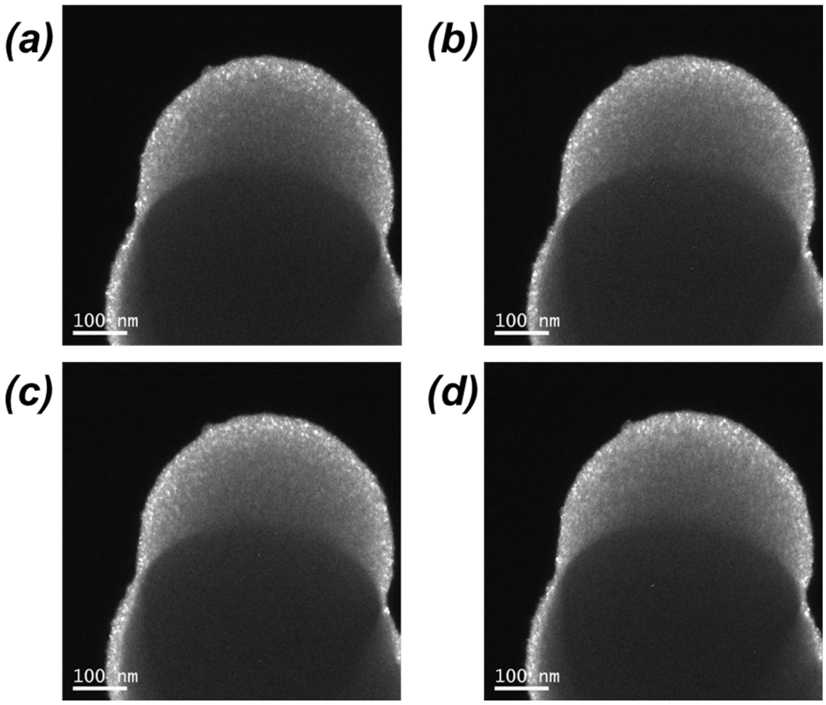

Figure S8: HAADF-STEM images of as-synthesised of FMS-TiO_2 spheres (2 M HNO_3 solution in ethanol/162 mM of TTIP as precursor; 1 min MW treatment) taken at: (a) 0°, (b) 90°, (c) 180° and (d) 270° of rotation.

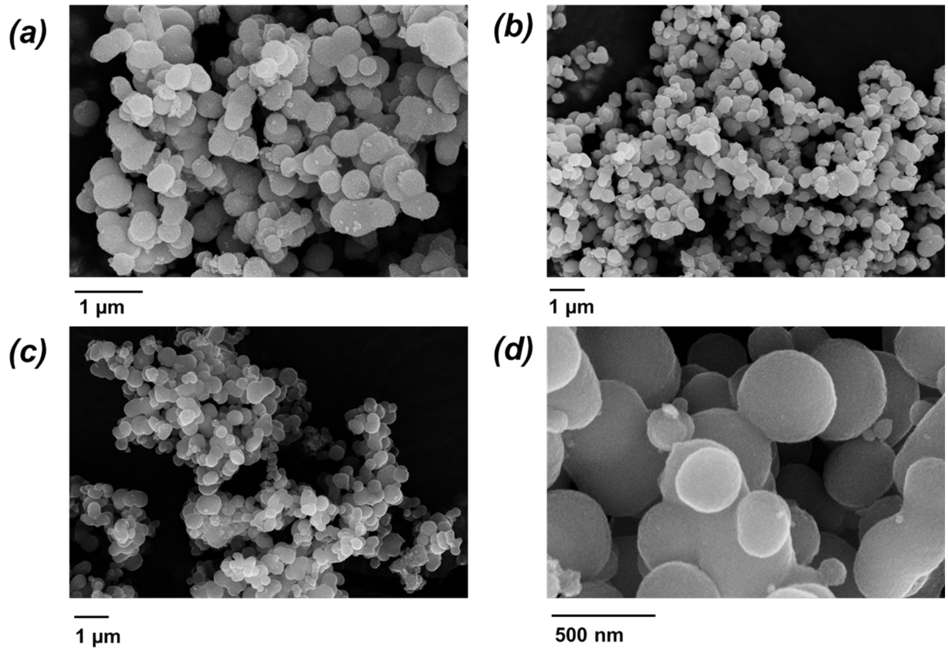

Figure S9: HR-SEM images showing examples of FMS TiO_2 sub-microspheres (2 M HNO_3 in ethanol/160 mM TTIP; 1 min MW irradiation time) following calcination: (a), (b) after 3 h of treatment at 300 °C; (c), (d) after 6 h treatment at 400 °C .

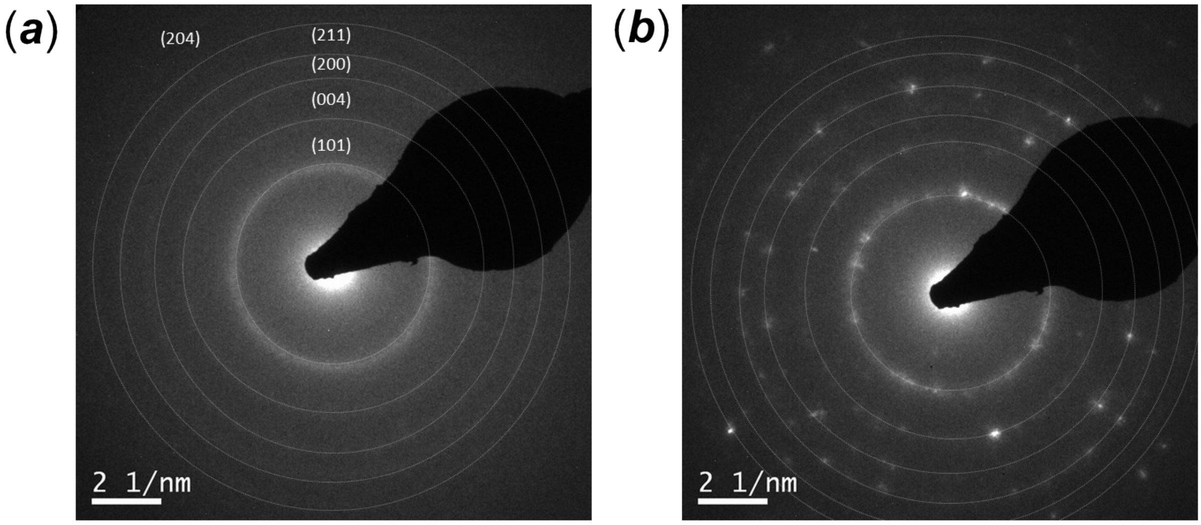

Figure S10: SAED images of: (a) untreated FMS TiO_2 sub-microspheres (2 M HNO_3 in ethanol/ 162 mM TTIP/ 1 min MW irradiation time) and (b) the same sample after calcination at 500 °C for 3 h.

Table S2: Assignment of the Raman modes for the spectra shown in Figures 4 and 5 of the manuscript, illustrating the modification of the vibration frequencies and the Eg I mode peak broadening as a function of acid and precursor concentration.

**Concentration**		**$E_{g\ I}$ / cm^{-1}**	**$E_{g\ I}$ FWHM / cm^{-1}**	**A_{1g} / cm^{-1}**	**B_{1g} / cm^{-1}**	**$E_{g\ III}$ / cm^{-1}**
Acid	*0.5 M*	152.6	25.7	398.5	516.3	647.5
	1 M	152.4	24.2	396.8	517.0	647.0
	2 M	151.0	22.8	398.1	518.4	644.7
Precursor	*20 mM*	152.9	65.1	422.9	519.9	626.0
	40 mM	152.9	41.4	409.8	515.9	636.7
	80 mM	147.4	25.7	393.7	510.9	636.9
	160 mM	147.9	22.9	392.7	514.4	640.0
	320 mM	148.3	23.3	394.5	514.5	642.3
	650 mM	148.3	24.3	392.4	512.1	639.2

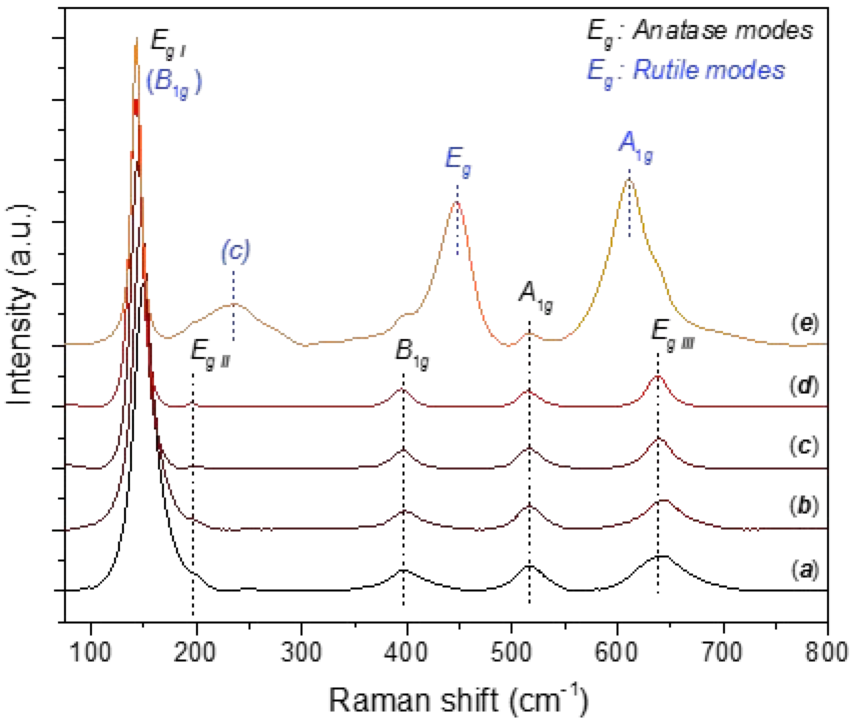

Figure S11: Raman spectra of FMS TiO_2 sub-microparticles (2 M HNO_3/162 mM TTIP precursor concentration; 1 min MW treatment): (a) untreated particles and after calcination (3 h; 5 °C min^{-1} heating rate) at: (b) 300 °C, (c) 400 °C; (d) 500 °C; (e) 600 °C. Characteristic modes for anatase (black) and rutile (blue) phases are marked in the figure.

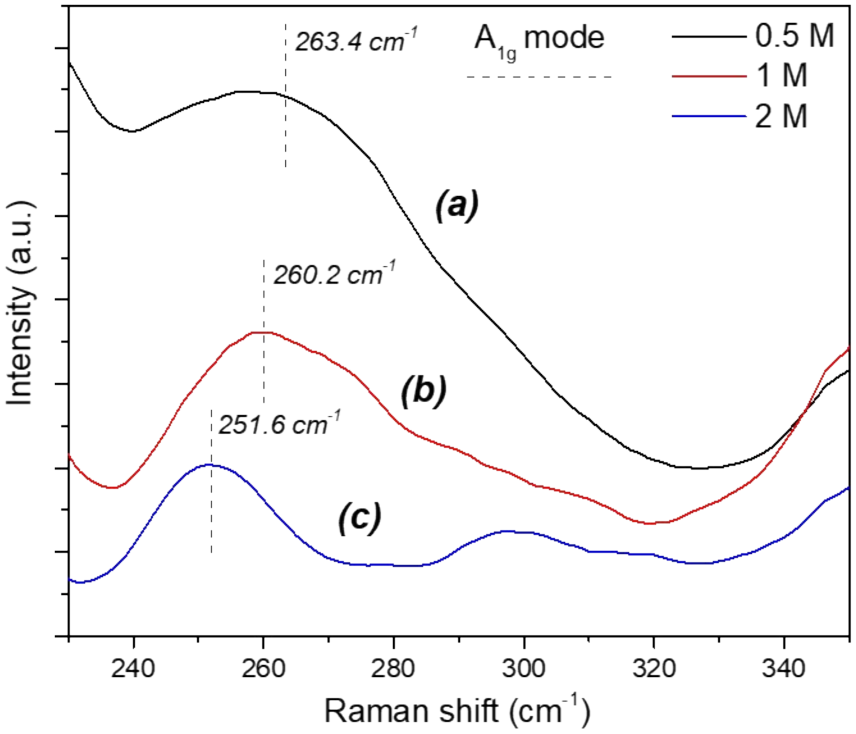

Figure S12: Magnification of the 240-340 cm^{-1} Raman spectral region for FMS TiO_2 sub-microsphere samples, prepared by using different HNO_3 concentration: (***a***) 0.5 M; (***b***) 1 M and (***c***) 2 M; all samples prepared with 160 mM TTIP and 1 min MW irradiation time. No anatase or rutile modes are present in this region and a band from the A_{1g} Raman mode associated with the Brookite phase of TiO_2 can be identified (dashed line).

Table S3: Specific surface area (SSA), pore size distribution, crystallite size (as estimated by the Scherrer equation), BET diameter and sub-microsphere diameter (from SEM) for selected FMS TiO_2 sub-microsphere samples. (The MW irradiation time was set at 1 min for all samples).

**[HNO_3], [TTIP] / M**	**Isotherm type**	**BET SSA / $m^2 g^{-1}$**	**BJH pore radius / Å**	**BJH pore vol / $cm^3 g^{-1}$**	**Crystallite[a] size / nm**	**BET diameter[b] / nm**	**SEM diameter / nm**
**0.5, 0.08**	I	251.5 ± 20.6	14.9 ± 3.7	0.022 ± 0.007	3.5	6.1 ± 0.5	1165 ± 379†
**0.5, 0.32**	I	159.7 ± 15.4	17.5 ± 2.4	0.060 ± 0.011	2.7	9.6 ± 0.9	1492 ± 566†
**1, 0.02**	II	55.7 ± 6.2	14.8 ± 3.9	0.014 ± 0.006	4.6	27.6 ± 1.6	265 ± 59
**1, 0.04**	II	106.2 ± 12.1	14.9 ± 3.9	0.049 ± 0.009	3.8	16.4 ± 1.7	264 ± 59
**1, 0.16**	I	214.2 ± 13.6	16.6 ± 2.5	0.014 ± 0.005	5.6	7.2 ± 0.5	730 ± 273
**1, 0.65**	I	251.1 ± 15.0	15.9 ± 1.9	0.011 ± 0.002	3.0	6.1 ± 0.4	1308 ± 740
**2, 0.08**	IV	510.1 ± 31.5	17.9 ± 3.5	0.642 ± 0.045	4.7	3.0 ± 0.2	294 ± 93
**2, 0.16**	IV	369.4 ± 17.4	17.5 ± 3.1	0.154 ± 0.011	3.8	4.2 ± 0.2	496 ± 230
**2, 0.65**	IV	507.4 ± 28.4	17.9 ± 2.7	0.316 ± 0.028	3.7	3.0 ± 0.2	672 ± 230

[a] ± 0.1 nm; [b] estimated from surface area assuming spherical particles with homogeneous size distribution; †multimodal distribution.

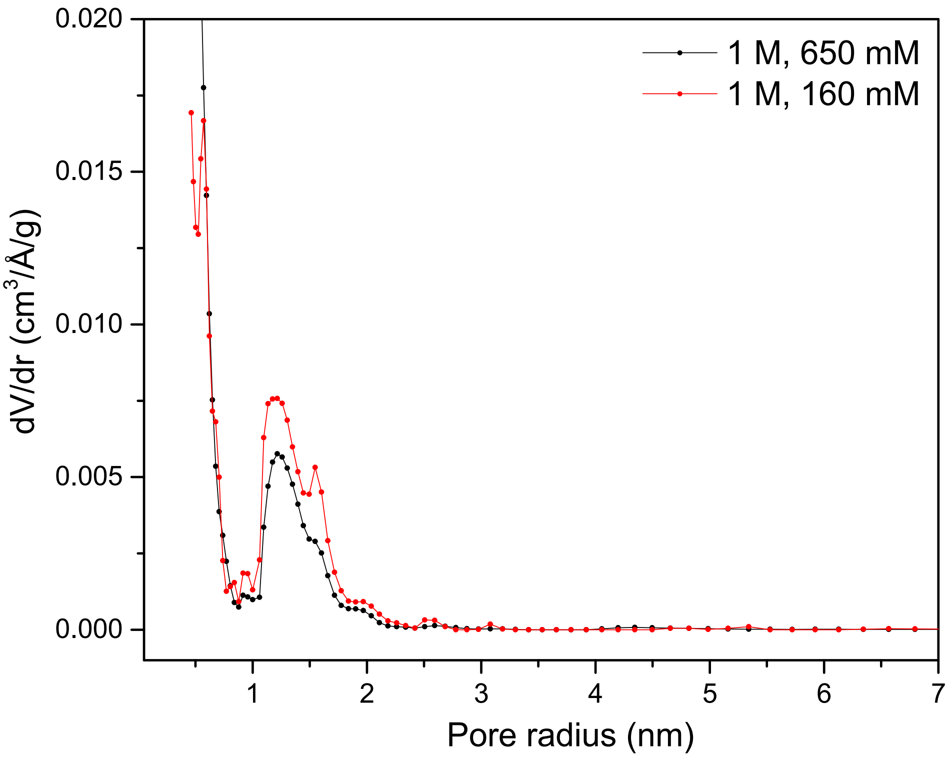

Figure S13: Pore size distribution of FMS TiO_2 sub-microspheres (1 M HNO_3/160 mM TTIP; 1 min MW treatment - red and 1 M HNO_3/650 mM TTIP; 1 min MW treatment - black) as calculated by the Quasi-Solid Density Functional Theory (QSDFT) method.

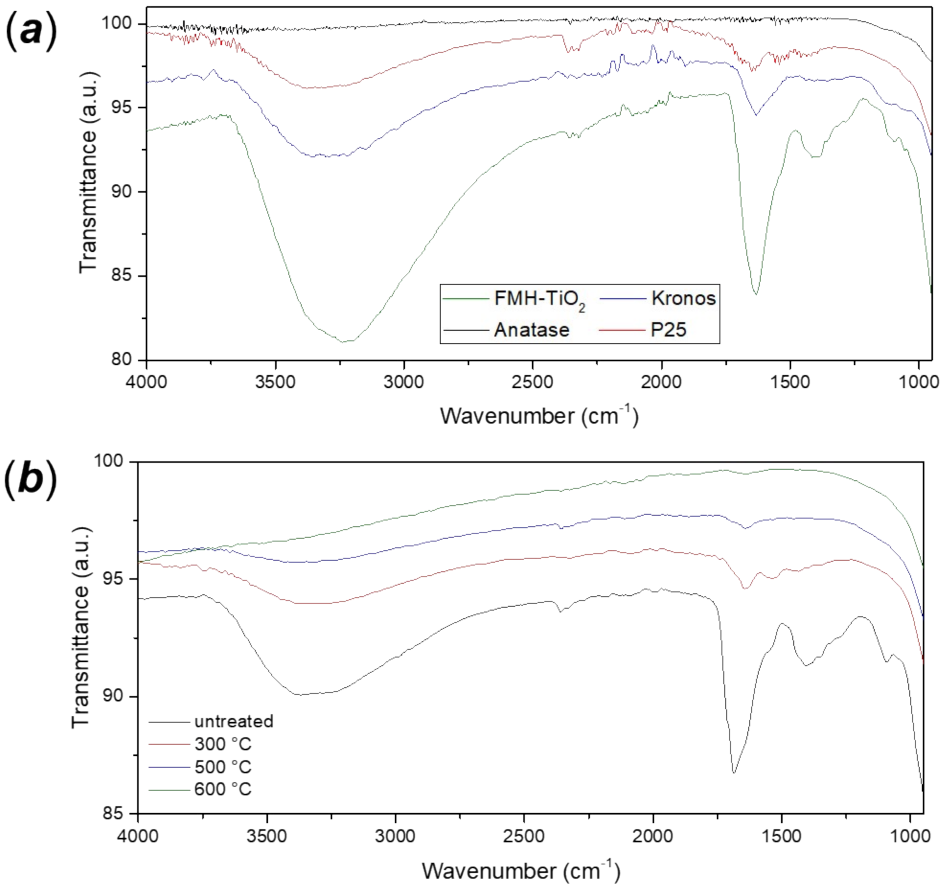

Figure S14: (a) IR spectrum of a representative sample of FMS TiO_2 sub-microspheres (2M HNO_3/160 mM TTIP ; 1 min MW treatment - green) as compared with commercial anatase (black) and the commercial TiO_2-based catalysts, P25 (red) and Kronos (blue); (b) IR spectra of FMS TiO_2 sub-microspheres (2M HNO_3/160 mM TTIP ; 1 min MW treatment) calcined for 3 h at different temperatures (untreated – black; 300 °C – red; 500 °C – blue; 600 °C – green).

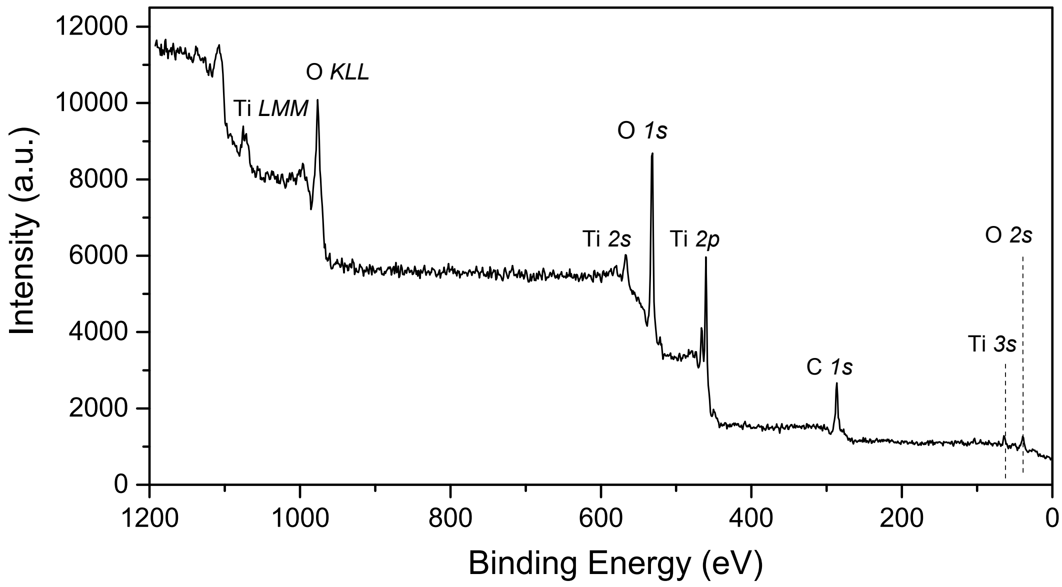

Figure S15: Complete XPS spectra of a representative sample of FMS TiO_2 sub-microspheres (2 M HNO_3/162 mM TTIP; 1 min MW irradiation time).

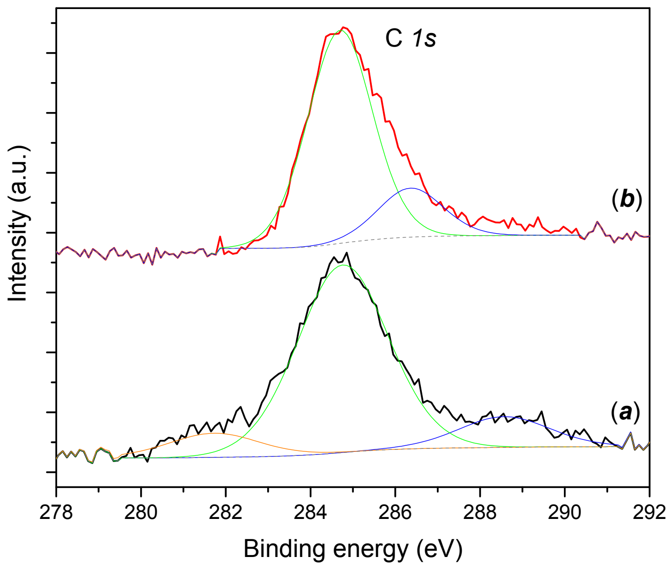

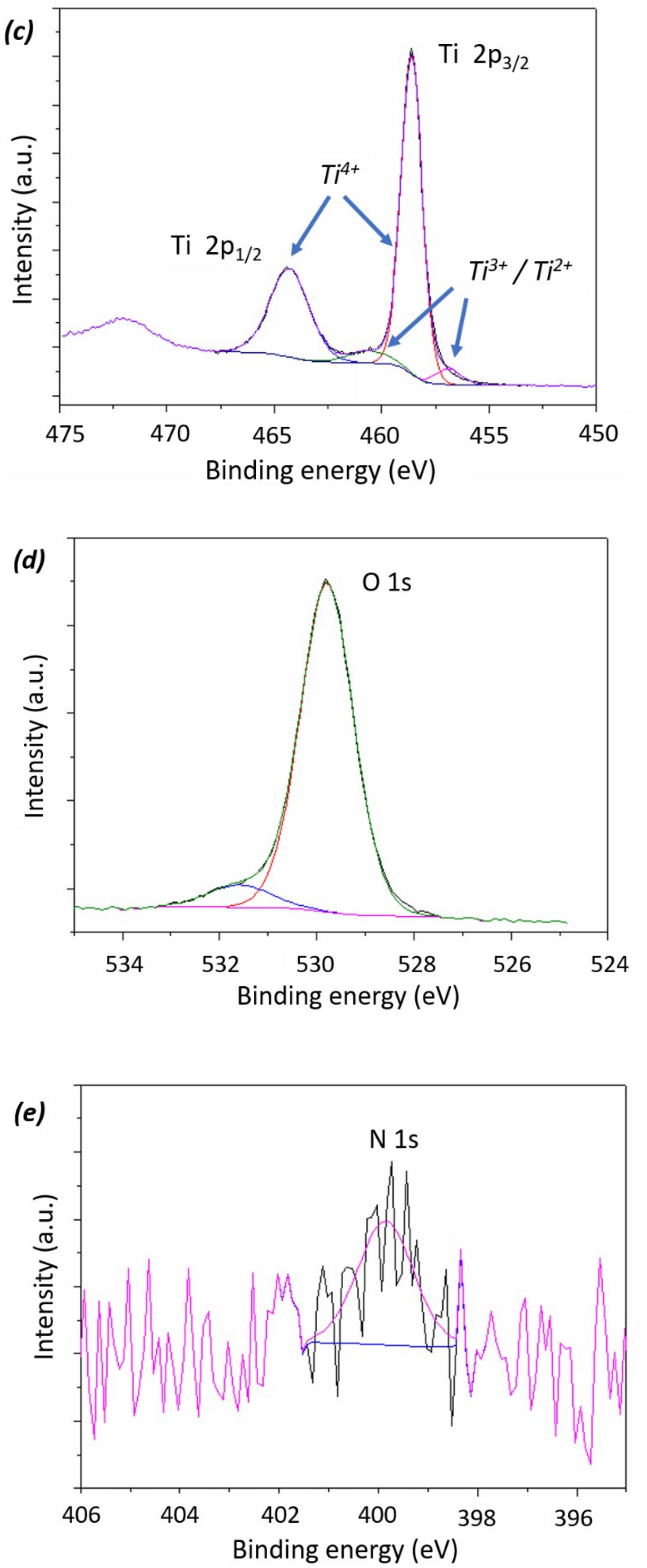

Figure S16: Comparison between: (a) the XPS C 1s signal of FMS TiO_2 sub-microspheres (2 M HNO_3/160 mM TTIP; 1 min MW treatment) and (b) the analogous signal from a sample of Aeroxide P25 (after thermal treatment at 500 °C for other research purposes); Fitted narrow scan XPS spectra of: (c) Ti 2p; (d) O 1s and (e) N 1s peaks from the same sample of FMS TiO_2 sub-microspheres shown in (a).

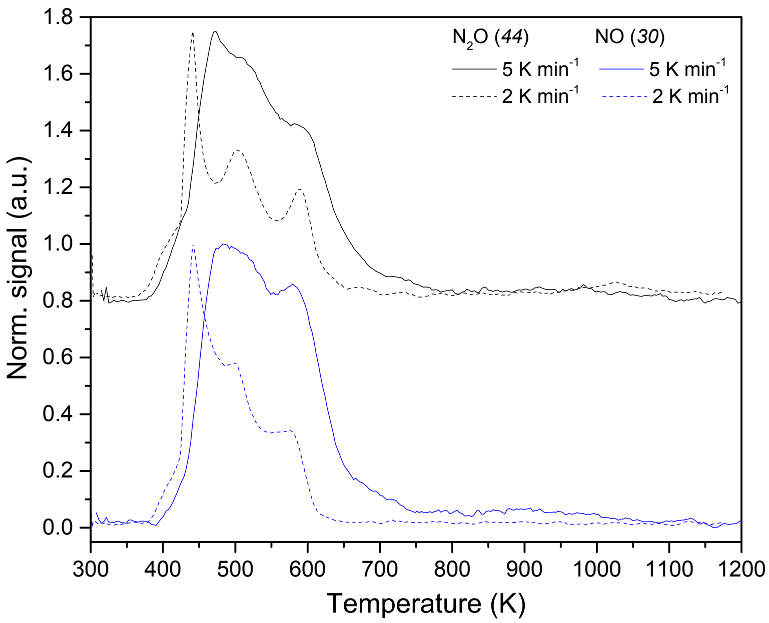

Figure S17: Evolved gas MS analysis of the signals tentatively assigned to N_2O (m/z = 44) and NO (m/z = 30) for a selected FMS-TiO_2 sub-microsphere sample (2 M HNO_3/324 mM TTIP; 1 min MW irradiation time) as a function of temperature and at different heating rates.

Table S4: C, H, N combustion microanalysis for selected FMS TiO_2 sub-microspheres (1 min MW irradiation time for all samples).

**Sample**	**Elements / wt.%**		
**[HNO_3], [TTIP] / M**	**C**	**H**	**N**
2, 0.64	2.32 ± 0.09	0.96 ± 0.04	1.32 ± 0.12
2, 0.16	3.09 ± 0.14	1.20 ± 0.05	0.78 ± 0.06
1, 0.08	2.11± 0.00	1.43 ± 0.07	0.17 ± 0.00
0.5, 0.080	3.37± 0.06	2.28 ± 0.15	1.07 ± 0.07
1, 0.040	1.94 ± 0.07	2.09 ± 0.07	0.17 ± 0.00

Table S5: Elemental content of FMS TiO_2 sub-microspheres (2 M HNO_3 solution/ 160 mM TTIP ; 1 min MW treatment) by XPS analysis (at.%) at the surface and at depths of 1 and 10 nm (by Ar etching).

	Elemental content / at.%		
**Element peak**	**Surface**	**Depth profile at 1 nm**	**Depth profile at 10 nm**
Ti 2p	15.15	18.86	20.93
O 1s	47.70	56.74	58.74
C 1s	37.15	23.57	20.00
N 1s	-	0.83	0.33

Table S6: Examples of experimental indirect and direct band gap values as a function of the synthesis conditions of selected FMS TiO_2 sub-microspheres (1 min MW irradiation time for all the samples).

	**[HNO_3] / M**	**[TTIP] / M**	**Indirect band gap / eV**	**Direct band gap / eV**
Anatase			3.23	3.47
FMS sub-microspheres	0.5	0.08	3.33-3.36	3.65-3.68
		0.16	3.29-3.31	3.68-3.70
		0.32	3.24-3.27	3.62-3.68
	1	0.04	3.45	3.76-3.80
		0.08	3.37-3.40	3.72-3.74
		0.16	3.27-3.35	3.67-3.73
	2	0.04	3.41-3.42	3.75-3.76
		0.08	3.41	3.77
		0.16	3.35-3.41	3.72-3.75
		0.32	3.33-3.36	3.66-3.70
		0.66	3.33	3.67

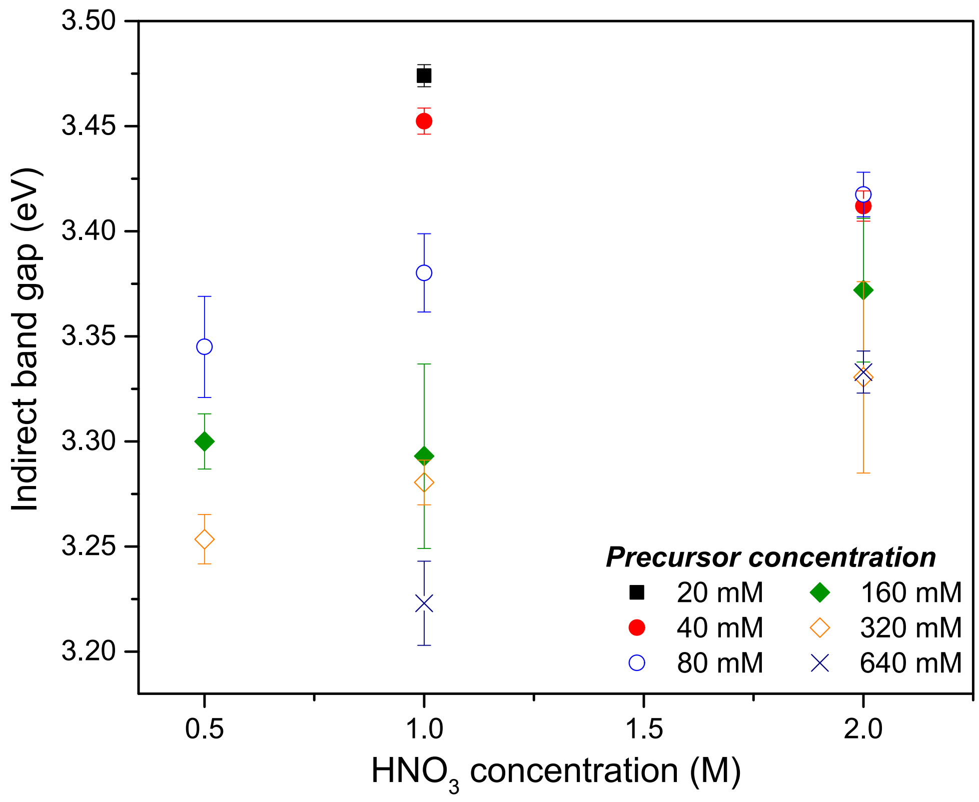

Figure S18: Indirect band gap as a function of the acid concentration for the synthesis of FMS TiO_2 sub-microspheres. The data are correlated with the precursor (TTIP) concentration used for the preparation of the TiO_2 particle. All samples were subjected to 1 min of MW irradiation time.

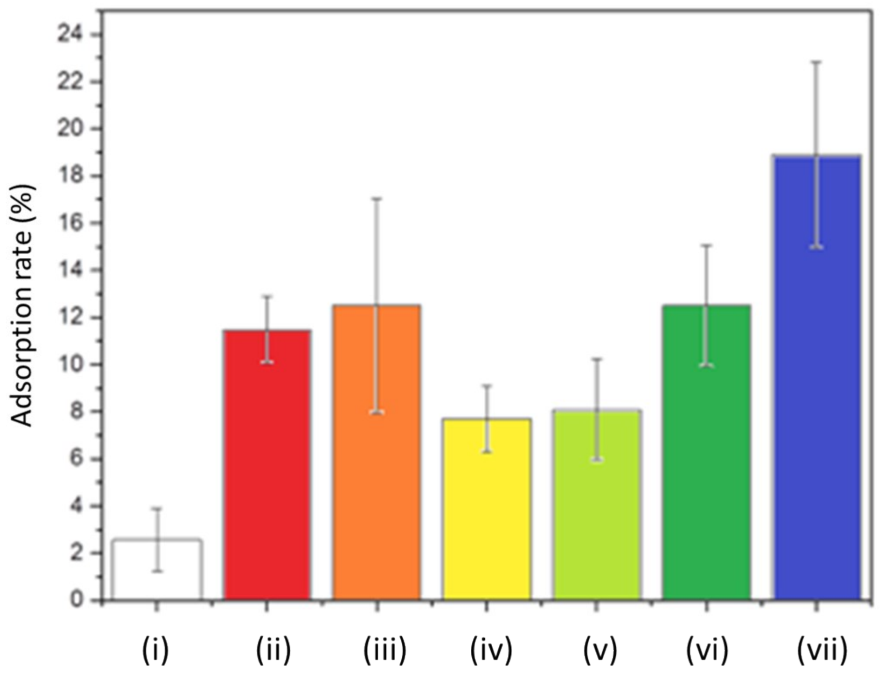

Figure S19: Initial adsorption for selected as-synthesised FMS-TiO_2 samples as compared to: (i) Aeroxide P25; (ii) 1M HNO_3/40 mM TTIP; (iii) 1M HCl/160 mM TTIP; (iv) 2M HNO_3/160 mM TTIP; (v) 2M HNO_3 (in water)/160 mM TTIP; (vi) 1M HNO_3/80 mM TTIP; (vii) 0.5M HNO_3/80 mM TTIP. All samples were subjected to 1 min of MW irradiation time.

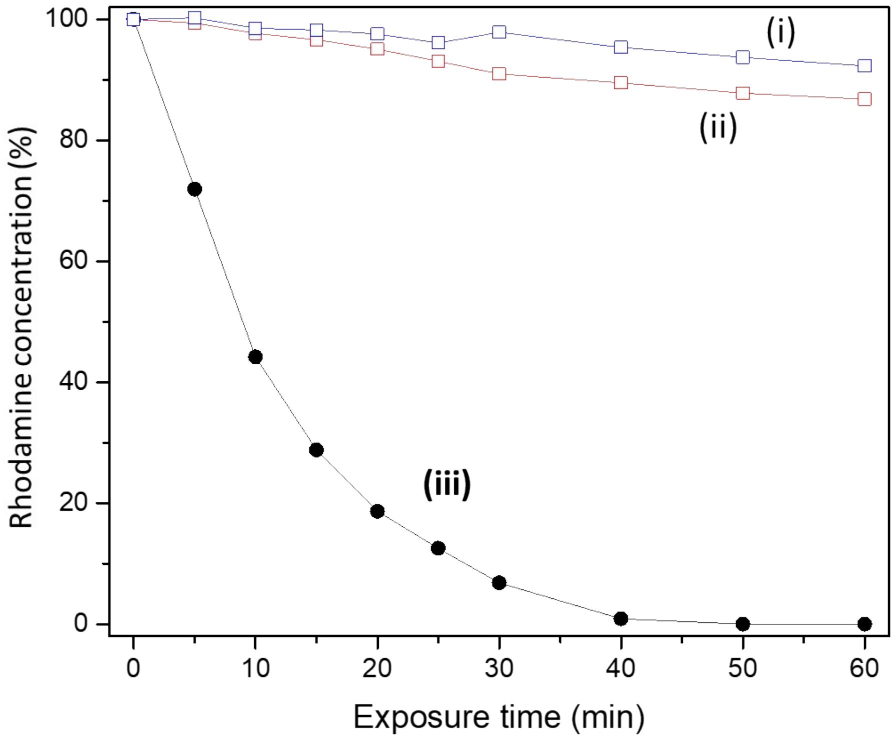

Figure S20 Degradation curves for selected FMS-TiO_2 sub-microsphere samples under UVA light: (i) 1M HNO_3/ 40 mM TTIP and (ii) 2M HNO_3/320 mM TTIP (1 min MW irradiation time for both samples) as compared to: (iii) Aeroxide P25.

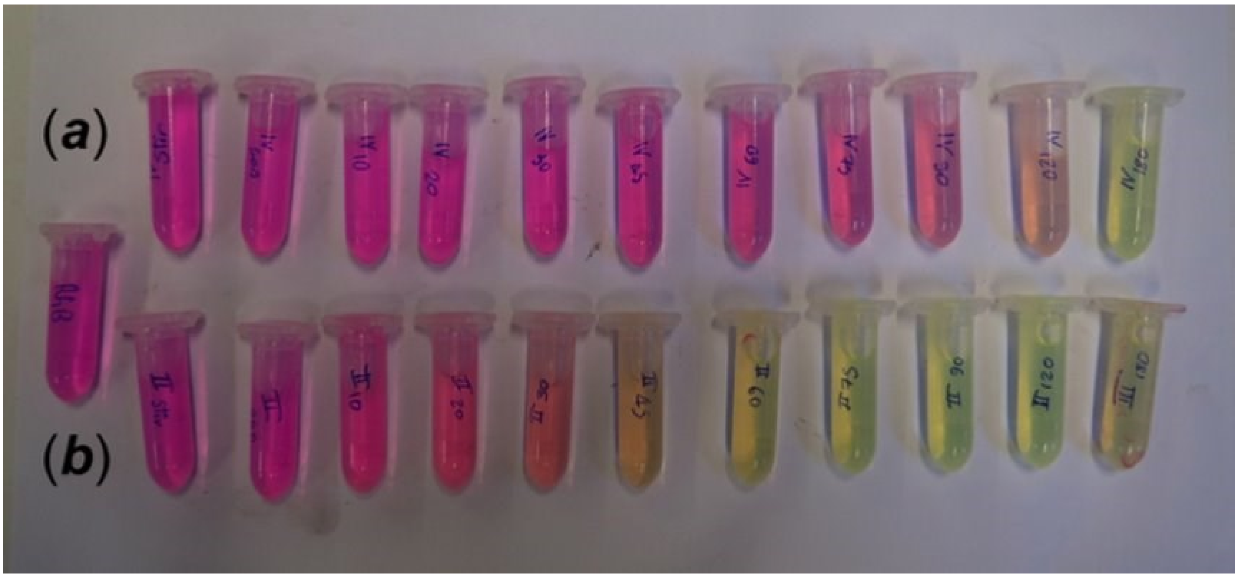

Figure S21: Image of the appearance of Rhodamine solution samples following degradation experiments performed using selected FMS TiO_2 sub-microspheres under visible light: (a) 0.5M HNO_3/160 mM TTIP and (b) 1M HNO_3/40 mM TTIP. All samples were subjected to 1 min of MW irradiation time.

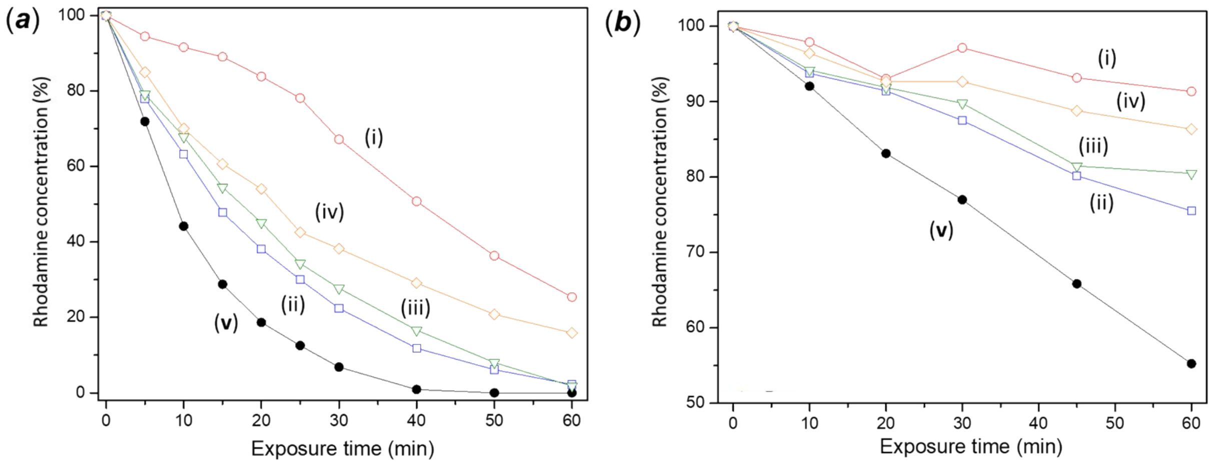

Figure S22: Degradation curves under: (a) UVA and (b) visible light for selected FMS TiO_2 sub-microspheres calcined in air (3h, 10 °C min^{-1}) at different calcination temperatures. The samples shown are: (i) 2M HNO_3/160 mM TTIP/400 °C, (ii) 2M HNO_3/160 mM TTIP/500 °C, (iii) 2M HNO_3/160 mM TTIP/300 °C; (iv) 2M HNO_3,320 mM TTIP/500 °C; (v) Aeroxide P25 (included as a comparison). All samples were subjected to 1 min of MW irradiation time.

Table S7: Zeta potential results taken from dispersions of FMS TiO_2 sub-microspheres produced under different synthesis conditions (all samples were prepared using 160 mM of TTIP as precursor and 2 M HNO_3).

**Synthesis parameters** ***(solvent/MW irradiation time/as made or calcination temperature)***	**Dispersion Concentration / mg L^{-1}**	**Zeta Potential / mV**	**Mobility / µm•cm V s^{-1}**	**Conductivity / mS cm^{-1}**
Ethanol/30s/as made	10mg/L	-28.57	-2.24	0.01
Ethanol/30s/as made	50mg/L	-25.40	-1.99	0.03
Ethanol/1 min/as made	1mg/L	-35.13	-2.75	0.01
Ethanol/1 min/as made	10mg/L	-27.97	-2.19	0.02
Ethanol/1 min/as made	50mg/L	-23.33	-1.83	0.02
Ethanol/1 min/300 °C	10mg/L	-27.73	-2.18	0.01
Ethanol/1 min/400 °C	10mg/L	-17.84	-1.40	0.02
Ethanol/1 min/500 °C	10mg/L	-16.02	-1.26	0.02
Water/1 min/ as made	10mg/L	-23.97	-1.88	0.02
Water/1 min/as made	50mg/L	-19.03	-1.49	0.01
Water/1 min/300 °C	50 mg/L	-23.78	-1.86	0.02
